# Substrate promiscuity of Dicer toward precursors of the let-7 family and their 3′-end modifications

**DOI:** 10.1007/s00018-023-05090-2

**Published:** 2024-01-23

**Authors:** Gunjan Dadhwal, Hebatallah Samy Saad, Jonathan Bouvette, Fatima El-Azzouzi, Pierre Dagenais, Pascale Legault

**Affiliations:** 1 Département de biochimie et médecine moléculaire, Université de Montréal, Downtown Station, Box 6128, Montreal, QC H3C 3J7 Canada; 2 Present Address: Molecular Biology Department, Guyot Hall, Princeton University, Princeton, NJ 08544 USA; 3 Present Address: Biochemistry Department, Wake Forest Biotech Place, 575 Patterson Avenue, Winston-Salem, NC 27101 USA

**Keywords:** Let-7 microRNA, miRNA precursors, RNA structural probing, Dicer binding, Dicer cleavage, TRBP, RNAse III enzyme, 3′-End processing

## Abstract

The human let-7 miRNA family consists of thirteen members that play critical roles in many biological processes, including development timing and tumor suppression, and their levels are disrupted in several diseases. Dicer is the endoribonuclease responsible for processing the precursor miRNA (pre-miRNA) to yield the mature miRNA, and thereby plays a crucial role in controlling the cellular levels of let-7 miRNAs. It is well established that the sequence and structural features of pre-miRNA hairpins such as the 5′-phosphate, the apical loop, and the 2-nt 3′-overhang are important for the processing activity of Dicer. Exceptionally, nine precursors of the let-7 family (pre-let-7) contain a 1-nt 3′-overhang and get mono-uridylated in vivo, presumably to allow efficient processing by Dicer. Pre-let-7 are also oligo-uridylated in vivo to promote their degradation and likely prevent their efficient processing by Dicer. In this study, we systematically investigated the impact of sequence and structural features of all human let-7 pre-miRNAs, including their 3′-end modifications, on Dicer binding and processing. Through the combination of SHAPE structural probing, in vitro binding and kinetic studies using purified human Dicer, we show that despite structural discrepancies among pre-let-7 RNAs, Dicer exhibits remarkable promiscuity in binding and cleaving these substrates. Moreover, the 1- or 2-nt 3′-overhang, 3′-mono-uridylation, and 3′-oligo-uridylation of pre-let-7 substrates appear to have little effect on Dicer binding and cleavage rates. Thus, this study extends current knowledge regarding the broad substrate specificity of Dicer and provides novel insight regarding the effect of 3′-modifications on binding and cleavage by Dicer.

**Supplementary Information:**

The online version contains supplementary material available at 10.1007/s00018-023-05090-2.

## Introduction

MiRNAs (miRNAs) are ~ 22 nucleotides (nt) small non-coding RNAs that regulate gene expression in nearly all biological processes [1–4]. They contain a seed sequence of 2–7 nt near the 5′ end that generally binds to the complementary region of target mRNAs to mediate translational repression and mRNA degradation [5, 6]. The lethal-7 (let-7) miRNAs are one of the largest families of miRNAs and are highly conserved across different species from worms to humans [7–9]. In humans, there are 10 mature let-7 miRNAs (let-7a, let-7b, let-7c, let-7d, let-7e, let-7f, let-7g, let-7i, miR-98, and miR-202) that share the same seed sequence, but are derived from 13 distinct gene loci [10]. They play critical roles in many biological processes including cell differentiation and development, and generally function as tumor suppressors [11, 12]. Alterations in the levels of let-7 miRNAs have been associated with several human diseases, such as several types of cancer, neurodegenerative disorders, and viral infections [8, 11, 13].

Let-7 miRNAs undergo the canonical pathway of maturation, which involves their processing by two endoribonucleases belonging to the RNase III family, Drosha and Dicer [12]. Drosha interacts with a dimer of DiGeorge critical region 8 (DGCR8) to form the Microprocessor complex that cleaves the primary miRNA (pri-miRNA) transcripts to a precursor miRNA (pre-miRNA) in the nucleus [9, 10]. The pre-miRNA is exported to the cytoplasm and therein processed by Dicer and its optional cofactor, the transactivation response element RNA-binding protein (TRBP), generating a miRNA duplex, from which the mature miRNA strand is selected as part of the RNA-induced silencing complex (RISC). Faulty processing of immature forms of miRNA (pri-miRNA and pre-miRNA) could generate non-cognate substrates for the subsequent steps and affect gene silencing [14]. Thus, the expression and the specific functions of miRNAs are highly dependent on both Drosha and Dicer processing [15].

Dicer is responsible for the second cleavage step of the canonical miRNA maturation pathway. It is a large multidomain protein that adopts an L-shape structure where the Platform-PAZ-Connector (PPC) cassette is positioned at the top of the “L,” the helicase domain forms the base and the two RNase III domains (RIIIDa and RIIIDb) fall in between [16–22]. This architecture allows Dicer to interact with the extremities of the pre-miRNA hairpins. The Platform and PAZ domains contain binding pockets for the 5′-phosphate and the 2-nt 3′-overhang of the pre-miRNA, respectively, and the helicase domain specifically interacts with the single-stranded hairpin loops of pre-miRNAs [23–25]. The RIIIDa and RIIIDb domains intramolecularly dimerize to form the catalytic center that cleaves each strand of the pre-miRNA. The anchoring of both ends of the pre-miRNA is critical for determining the cleavage sites of Dicer as it locates the catalytic center ~ 21–22 nt or ~ 58 Å away from the free ends of the pre-miRNAs [19].The dsRBD domain has RNA-binding affinity and enhances the cleavage efficiency of Dicer. Structural investigations along with biochemical and functional studies have revealed that these specific interactions of different domains of Dicer with the pre-miRNA are critical for precise and efficient processing [26]. The most recent cryo-EM studies have further supported the importance of these interactions by capturing structural rearrangements that occur during the transition from a pre-dicing state to a dicing state in both human and mouse Dicer [20–22]. These studies reveal that the catalytic center of Dicer is far from the pre-miRNA cleavage sites in the pre-dicing state and that Dicer undergoes structural rearrangements to accommodate the RNA substrate at the catalytic center.

Several sequence and structural features of pre-miRNAs are known to be associated with the processing efficiency of Dicer. Biochemical studies have shown that Dicer preferentially bind and cleave pre-miRNAs with a 2-nt 3′-overhang and apical loops > 9 nt [27]. It requires the 5′-phosphate for precise cleavage and altering the length of the 3′-overhang impairs processing by Dicer [25]. The ends of the pre-miRNA and the position of the apical loop are important for precise cleavage by Dicer [28, 29], exemplified by the numerous “counting rules” described in the literature. Dicer can determine the cleavage site based on a 5′ counting rule (21–22 nt away from the 5′-phosphate), a 3′ counting rule (21–22 nt from the 3′-end), and/or a loop counting rule (2-nt away from the apical loop) [27, 30–32]. The mismatches, bulges, symmetrical and asymmetrical internal loops in the double-stranded RNA (dsRNA) region of the pre-miRNAs have been associated with processing efficiency of Dicer, although not in all cases [29, 33]. However, specific structures at the Dicer cleavage site (e.g., bulge, base triplet) are known to affect Dicer cleavage [33, 34]. The sequence identity two nucleotides upstream and downstream of the cleavage site (5′-NN^NN-3′) has also been found to modulate Dicer cleavage [35, 36]. In addition, a paired guanine (G) followed by a pyrimidine (Y) and a mismatched cytosine or adenine (M) (GYM motif) near the cleavage site promotes Dicer processing at a specific position [37]. Thus, several structural features of pre-miRNA regulate miRNA maturation pathway by modulating the cleavage activity of Dicer. However, the roles of different structural features for Dicer processing of the let-7 family have not been investigated systematically. Moreover, the secondary structures of let-7 precursors have been previously determined for only a few family members [19, 38, 39]. Given that differences in the structure of pre-miRNA may modulate Dicer activity, it is important to further examine the secondary structures of all let-7 precursors.

Several RNA-binding proteins (RBPs), such as Lin28A/B and terminal uridyltransferases (TUTase) also known as terminal nucleotidyltransferases (TENTs), add another layer of regulation in pre-let-7 processing by Dicer. By themselves, TUTases interact transiently with pre-let-7 and can add 1 to 3 uridines at the 3′-end of pre-let-7 [40–42]. However, in the presence of Lin28, TUTases (TENT2 also known as TUT2, TUT4, or TUT7) were found to oligo-uridylate pre-let-7, and this modification is known to promote degradation by the 3′–5′ exonuclease DIS3L2 [41, 43–45]. In the pre-let-7 family, 9 members contain a 1-nt 3′-overhang (Group II pre-miRNA) instead of a typical 2-nt 3′-overhang (Group I pre-miRNA) found in most pre-miRNAs. These group II precursors get mono-uridylated in vivo by TUTases to generate a 2-nt 3′-overhang [41]. About 20–30% of Group II pre-let-7 in HEK-293 cells are found to contain an extra uridine at the 3′-end and 1% contain adenine [41, 46, 47]. The mono-uridylated pre-let-7 have been shown to be processed more efficiently by an immuno-purified Dicer than the unmodified pre-let-7 [41], presumably because they are better substrates for Dicer binding and cleavage. Although oligo-uridylated pre–let-7 are viewed as being less responsive to processing by Dicer [40], it is not clear how oligo-uridylation directly affects pre-miRNA cleavage by Dicer. In addition, TENT2 can add adenines at the 3′-end of pre-miRNAs, which have been shown to affect miRNA maturation [47, 48]. However, it is not clear how these 3′-end modifications of pre-let-7 affect Dicer processing.

In this study, the secondary structural features of all members of the let-7 family were systematically characterized by Selective 2′-Hydroxyl Acylation analyzed by Primer Extension (SHAPE) and detailed thermodynamic and kinetic investigations were performed in vitro using purified recombinant Dicer and pre-let-7 RNAs. Surprisingly, this study reveals that Dicer shows remarkable promiscuity for structurally different substrates, since it binds with similar affinity and cleaves with similar specificity all pre-let-7 substrates, including those of Group II with a 1-nt 3′-overhang. We also investigated the effect of 3′-end modifications of pre-let-7 in modulating binding and cleavage by Dicer. This study provides evidence that mono-uridylation does not substantially affect the rate of cleavage by Dicer in vitro and brings new information on the effect of 3′-extension on Dicer activity. Overall, this study challenges current views regarding the effect of 3′-end modifications on the processing activity of Dicer while highlighting its remarkable promiscuity toward pre-let-7 substrates.

## Material and methods

### Cloning

For this study, human pre-let-7 sequences were retrieved from the miRBase [49], and corrected as needed using results from Drosha cleavage assays of pre-let-7 RNAs [41]. For SHAPE studies, the coding sequence for each pre-let-7 RNA was cloned into a pTZ19R vector containing a cassette composed of a 5′-linker, a 3′-linker, and a reverse transcription (RT) primer binding site, as previously described [50]. Based on mFold secondary structure predictions [51], a one-nucleotide modification at the + 1 position of the 3′-linker sequence was introduced, changing the U into G, to prevent interaction of the cassette with the pre-let-7 RNAs. For Dicer binding and cleavage studies, the pUC19-HH-pre-let-7-HDV vectors used for pre-let-7 transcription were constructed as described previously for pre-let-7a-1 [52].

### RNA synthesis and purification

For SHAPE probing, the linearized plasmid was incubated with T7 RNA polymerase (prepared in house) for 3 h at 37 °C in the presence of 40 mM Tris pH 7.6, 50 mM DTT, 30 mM MgCl_2_, 1 mM spermidine, 0.1% Triton X-100, 4 mM for each standard NTP, and 0.2 U/uL RNasin ribonuclease inhibitor. The transcribed RNA was purified using 10% denaturing polyacrylamide gels, visualized by UV shadowing, and excised from the gel. The RNA was then extracted from the gel by crush and soak in TEN buffer (0.3 M NaCl, 10 mM Tris and 1 mM EDTA, pH 7.6); the eluate was filtered, ethanol precipitated and resuspended in ddH_2_O. The RNA purity was assessed on 10% polyacrylamide denaturing gels.

For Dicer binding and kinetic studies, the synthesis of all pre-let-7 substrates and 5′-end phosphorylation were carried out as described previously [52, 53]. Briefly, each pre-let-7 RNA was transcribed in vitro with 5′-Hammerhead and 3′-HDV ribozyme tags that self-cleaved in the transcription reaction to yield an RNA with homogeneous 5′- and 3′-ends [54]. The pre-miRNA was then purified by denaturing gel electrophoresis and treated with T4 polynucleotide kinase using either unlabeled ATP or ATP-γ-^32^P to mimic the 5′- and 3′-ends of natural Dicer substrates resulting from cleavage of primary miRNAs by Drosha. This enzymatic treatment phosphorylates the 5′-OH and modifies the 3′-end by opening the 2′,3′-cyclic phosphate and removing the 3′-phosphate [55, 56]. Subsequently, the 5′-monophosphorylated RNAs were purified either by denaturing anion-exchange HPLC (cold RNA) or by denaturing gel electrophoresis (^32^P-labeled RNA) and stored in TE buffer (10 mM Tris pH 8.0 and 1 mM EDTA) at − 20 °C.

### SHAPE probing

The secondary structure of let-7 RNA precursors was characterized by SHAPE. The chemical probing experiments were performed in triplicate for all pre-let-7 RNAs, except for pre-let-7a-3 and pre-let-7f-1, which were done in duplicate. Typically, 16 pmol of the RNA was refolded in 50 mM HEPES pH 7.4 and 50 mM NaCl, heated at 90 °C for 2 min, then cooled on ice for 10 min, after which 5 mM MgCl_2_ was added. The refolded RNA was then treated with 1 µL of 80 mM of 1-methyl-7-nitroisatoic anhydride (1M7) for 5 min at 37 °C in a final reaction volume of 12 µL. A control sample was prepared, in which DMSO was added to the RNA instead of the 1M7 reagent. Following the reaction, the RNA was ethanol precipitated and resuspended in ddH_2_O. Using this RNA, a reverse transcription (RT) reaction was performed according to the manufacturer’s protocol using 16 pmol of a 5′-6FAM-labeled primer (5′-CGA ACC GGA CCG AAG CCC G-3′) to generate 5′-labeled cDNA fragments. Using the same RT protocol, an RNA sequencing reaction was set up, with either didATPs, didTTPs or didCTPs, using a 1:1 dNTP:didNTP ratio. The cDNA fragments from RT and sequencing reactions were analyzed by capillary electrophoresis at the genomics platform of the Institute for Research in Immunology and Cancer (IRIC) at the Université de Montréal. The resulting SHAPE data were processed using the RiboCAT and RiboDOG software [57] to generate constraints files that were input into the RNAstructure software [58]. Using the Fold algorithm of the RNAstructure software, the secondary structures of let-7 RNA precursors were generated with default settings except for: Maximum Loop Size = 40; Maximum % energy difference = 50 (Δ*E*_max_ = 50%); SHAPE slope = 2.6; and SHAPE intercept = −0.8 [59].

### Protein expression and purification

The wild-type human Dicer (Dicer) and catalytically inactive variant (ciDicer: D1320A/D1709A) proteins were expressed and purified as described previously [52]. Briefly, the proteins were expressed from a pTT5 vector in HEK 293-6E cells grown in suspension. The cells were first diluted to 0.8 × 10^6^ cells/mL 24 h before transfection with the pTT5 vector along with 2× polyethylenimine (PEI). After three days, the cells were harvested, and the pellets were frozen with liquid nitrogen and stored at −80 °C until purification. Dicer proteins were purified by anion-exchange chromatography (Q Sepharose Fast Flow in a XK 26/20 column), Ni–NTA affinity chromatography (5-mL HisTrap High Performance column), and size-exclusion chromatography (Superdex 200 16/600 column), as described previously [52]. The last column was pre-equilibrated with storage buffer (50 mM Tris pH 8.2, 10 mM NaCl/KCl 24:1, 0.5 mM MgCl_2_, 0.5 mM TCEP, 5% Sucrose, and 0.3 mM DDM (*n*-dodecyl-β-d-maltoside)) and the eluted fractions containing monomeric Dicer were concentrated to around 2–3 µM on a 7-mL Apollo concentrator 150-kDa MWCO (Orbital Biosciences). The purified protein was aliquoted, frozen in liquid nitrogen, and stored at −80 °C.

For TRBP expression, a pET21a vector containing the TRBP sequence (DNASU HSCD00045566 [60]) was transformed in *Escherichia coli* Rosetta (DE3) pLys strain. The bacterial cultures were grown at 37 °C in LB medium supplemented with 35 mg/L chloramphenicol and 100 mg/L ampicilin, induced at an OD600 of 0.6 with 0.1 mM isopropyl-β-d-thiogalactopyranoside (IPTG), and then grown for 16 h at 16 °C. The cells were harvested by centrifugation and resuspended in HisA buffer (20 mM Tris pH 8.0, 500 mM NaCl, 20 mM imidazole, and 4 M Urea) supplemented with one tablet of Roche complete protease inhibitor. The cells were then lysed by French press and centrifuged at 100,000×*g* for 45 min at 4 °C. The supernatant was loaded onto a 20-mL gravity flow Nickel-bound IMAC column (GE Healthcare) equilibrated with HisA buffer. The column was then washed with 10 column volumes (CV) of HisA buffer, and the bound proteins eluted with HisB buffer (20 mM Tris pH 8.0, 500 mM NaCl and 300 mM imidazole). The fractions containing TRBP were pooled, supplemented with TEV protease and dialyzed overnight in TEV buffer (20 mM Tris pH 8.0, 100 mM NaCl and 5 mM DTT) at room temperature. The retentate was then loaded on 60-mL of SP Sepharose High Performance media packed in a XK 26/20 column (GE Healthcare) and equilibrated with SPsephA buffer (20 mM Tris pH 8.0 and 1 mM DTT). The column was then washed with 2.5 CV of 10% SPsephB buffer (20 mM Tris pH 8.0, 500 mM NaCl and 1 mM DTT), and TRBP was eluted using a linear gradient (from 10 to 100% over 4 CV) of SPsephB. Fractions containing TRBP were pooled and dialyzed in storage buffer (100 mM Tris pH 7.4, 100 mM NaCl and 10% glycerol). The dialyzed sample was then concentrated to 20–50 μM, aliquoted, flash frozen in liquid nitrogen, and stored at −80 °C.

### Binding assays for ***K***_d_ determination

Binding assays to characterize the pre-let-7/Dicer interactions were performed with ciDicer using an electrophoretic mobility shift assay (EMSA), as described previously [52]. Typically, the binding reactions were initiated by adding 10 µL of the RNA sample to 10 µL of the protein samples, both diluted with Dicer binding (DB) buffer (50 mM Tris pH 7.6, 50 mM NaCl, 10% glycerol, 0.05% NP-40, and 2 mM DTT) and incubated at 4 °C for 30 min. The RNA was heated at 95 °C for 2 min and snap-cooled on ice for 5 min before diluting it with the DB buffer. Dicer binding reactions used to determine *K*_d_ values contained 2 pM ^32^P-labeled RNA and protein concentrations varying between 0.01× and 100× of the estimated *K*_d_ value. The Dicer-binding reactions were loaded on a 4–15% gradient polyacrylamide gel (37.5:1 acrylamide:bis-acrylamide) in Tris–Glycine buffer (25 mM Tris–Base pH 7.4 and 200 mM glycine) and run for 2 h at 200 V in the cold room. The protein-bound and unbound RNA fractions were quantified by phosphor imaging using either a personal molecular imager (PMI) system (BioRad) or a GE Typhoon FLA 9500. The fraction of bound RNA was then plotted against protein concentration, and the binding data were fitted to the Hill equation with the OriginPro 2020 SR1 software (OriginLab). The *K*_d_ values are reported with experimental errors taken from the average and standard deviations from at least three independent experiments.

### Steady-state kinetic studies of pre-let-7 cleavage by Dicer

Steady-state kinetic studies involving Dicer and the pre-let-7 substrates were carried out as described previously [52]. Briefly, RNA and proteins solutions were prepared separately at twice their final desired concentration in 30 µL of the cleavage buffer (50 mM HEPES pH 7.4, 50 mM NaCl, 5 mM MgCl_2_ and 0.05% NP-40). The RNA solutions, containing 80 pM of ^32^P-labeled pre-let-7 substrate and different concentrations of the corresponding non-labeled 5′-monophosphorylated RNA (from 80 to 7680 nM), were heated at 95 °C for 2 min and snap-cooled on ice for at least 5 min to refold the RNA. Both RNA and protein solutions were pre-heated for 5 min at 37 °C, and cleavage reactions were initiated by adding 30 µL of the protein solution to 30 μL of the RNA solution. A 5-µL aliquot of the cleavage reaction was taken at 5, 10, 15, 20, 25, and 30 min, mixed with 25 µL of stop buffer and placed on ice. The cleavage reactions were analyzed by denaturing gel electrophoresis [15% acrylamide:bis-acrylamide (19:1)/7 M urea gel]. The amounts of substrate (*S*) and product (*P*) were quantified from radioactive bands detected by phosphor imaging, and the fraction of product [*F* = *P*/(*S* + *P*)] was plotted against time. The resulting time courses were fitted by linear regression (*y* = *mx *+ *b*); the slope (*m*) of the linear fit was taken as the initial velocity (*v*_0_). To ensure that initial cleavage rates were measured under steady-state conditions, the enzyme concentration ([*E*]) was varied along with the initial substrate concentration ([*S*]) to maintain [*S*]/[*E*] ≥ 50, and time points were collected after at least one turnover of the enzyme pool and with 5–10% of the substrate being cleaved within 30 min. The dependence of *v*_0_/[*E*] on [*S*] was plotted and fitted to the Michaelis–Menten equation (written as *v*_0_/[*E*] = *k*_cat_[*S*]/(*K*_M_ + [*S*]) with the OriginPro 2020 SR1 software (OriginLab) to derive *k*_cat_ and *K*_M_ values. The quality of the fit was obtained from the square of the correlation coefficient (*R*^2^), and in all cases *R*^*2*^ ≥ 0.97 and the distribution of residuals was random around the regression line.

### Single turnover kinetic studies of pre-let-7 cleavage by Dicer

Single turnover kinetic studies were typically carried out in 60-µL reaction volumes containing 0.1 nM ^32^P-labeled pre-let-7 substrate and 5 nM Dicer in cleavage buffer. The RNA was first refolded by heating at 95 °C for 2 min and snap-cooled on ice. Both RNA and protein-containing solutions were pre-heated for 5 min at 37 °C, and reactions were initiated by adding the RNA solution (2 µL) to the protein-containing solution. The cleavage was carried at 37 °C and 5-µL aliquots were taken at specific time points (0.5, 1, 2, 5, 10, 20, 30, 40, and 60 min). To stop the reaction, each aliquot was diluted in 45 µL of stop buffer (0.1% bromophenol blue, 95% formamide, and 50 mM EDTA) and immediately placed on ice. For the 0-min time point, no Dicer was added to the cleavage reaction. The samples were heated at 95 °C for 2 min before loading on a 15% acrylamide:bis-acrylamide (19:1)/7 M urea gel, that was then run at 220 V for 1 h. The gels were soaked in a buffer containing 20% ethanol, 5% acetic acid, and 1 × TBE for 30 min before they were dried with a vacuum gel dryer. The dried gel was then exposed to the phosphor imaging screen overnight and scanned using phosphor imaging. The amounts of substrate (*S*) and product (*P*) were quantified from radioactive bands in the gel and the percentage of cleavage [%*C *= P/(*S* + *P*) * 100] was plotted against time. The data was fit to an exponential equation using nonlinear regression in OriginPro 2020 SR1 software (OriginLab) by using the equation *y* = *y*_0_ + *A* * exp (−*R*_0_ * *x*), where *y* is the percentage of cleavage at a given time point, *y*_0_ is the maximum percentage of cleavage at the plateau (*x* ≥ ∞), and *R*_0_ is the observed rate constant (*k*_obs_). The quality of the fit was obtained from the square of the correlation coefficient (*R*^2^), and in all cases, *R*^2^ ≥ 0.98. The *k*_obs_ values are reported with experimental errors taken from the average and standard deviations from at least three independent experiments.

### Characterization of miRNA-5p products

To characterize the 5p products of pre-let-7 cleavage by Dicer, we carried out cleavage assays under single-turnover conditions as described above, but taking a single time point at 15 min. T1 ladders were generated by incubating 0.05 nM of ^32^P-labeled pre-let-7a-2 (or pre-let-7f-1) in a 10-µL volume containing 20 mM sodium citrate pH 5.0, 1 mM EDTA, 7 M Urea, 0.37 mg/mL yeast tRNA with 0.05 U of T1 nuclease (Sigma-Aldrich) for 5 min at 37 °C. The hydroxyl ladders were generated by incubating 1 nM of ^32^P-labeled pre-let-7a-2 (or pre-let-7f-1) in a 5-μL reaction volume containing 50 mM NaHCO_3_/Na_2_CO_3_ pH 9.2, 1 mM EDTA, 0.25 mg/mL yeast tRNA for 7 min at 95 °C. The reaction was stopped by adding ~ 1× volume of stop buffer. Each reaction was further diluted with 20 mL of stop buffer and analyzed by loading 10 μL of the diluted reaction on a 20% acrylamide:bis-acrylamide (19:1)/7 M urea gel run at 800 V for about 2.5 h. The dried gel was visualized using phosphor imaging.

### Pre-let-7 cleavage by Dicer in the presence of TRBP

Single-turnover kinetics for pre-let-7 cleavage by Dicer in the presence of TRBP was performed under standard conditions using 0.1 nM pre-let-7 substrate, 5 nM Dicer and 5 nM TRBP. For pre-let-7 cleavage by Dicer under multiple-turnover conditions in the presence of TRBP, the experiments were performed as described earlier, except that 25 nM pre-let-7 substrate, 0.5 nM Dicer, and 10 nM TRBP were used, and aliquots were taken at 5, 10, 20, 30, 40, 60, 90, and 120 min. For both single-turnover and multiple-turnover kinetic experiments, Dicer and TRBP were co-incubated at 37 °C for at least 10 min before adding RNA to initiate the cleavage reaction.

### Model building of the Dicer-pre-let-7 complex

To build the model of Dicer-TRBP bound to a pre-let-7 RNA with an extended 3′-end, the PDB file 5ZAL [19] was used as initial atomic coordinates. The 3′-end extension was then modeled as a single-stranded A-form helix using BIOVIA Discovery Studio Visualizer and energy minimized using CHARMM [61]. STRIDER was used for analysis of steric hindrance [62].

## Results

### Primary structure of the let-7 pre-miRNAs

A comparative analysis of pre-let-7 RNA sequences was first conducted based on previous studies to uncover their distinctive features beyond their classification as Group I or Group II pre-miRNAs (Supplementary Table S1). Their length ranges from 58 to 79 nt, with pre-miR-202 (m202) being the shortest and pre-miR-98 (m98) the longest. For twelve members, the conserved seed sequence is nested in the 5p-miRNA, whereas for m202, the conserved seed sequence is in the 3p-miRNA [10]. Dicer cleavage sites are fairly conserved for the 5p let-7, with UU^(G/U)N and (C/A)A^CU consensus at the 5p and 3p sites, respectively [49]. For miR-202, the cleavage sites are different with UG^AG and AA^AG at the 5p and 3p sites, respectively [49]. These Dicer cleavage sites separate the double-stranded miRNA portion (dsRNA) from the apical loop region, which ranges between 14 and 34 nt.

### Secondary structure of the let-7 pre-miRNAs

**Fig. 1:**
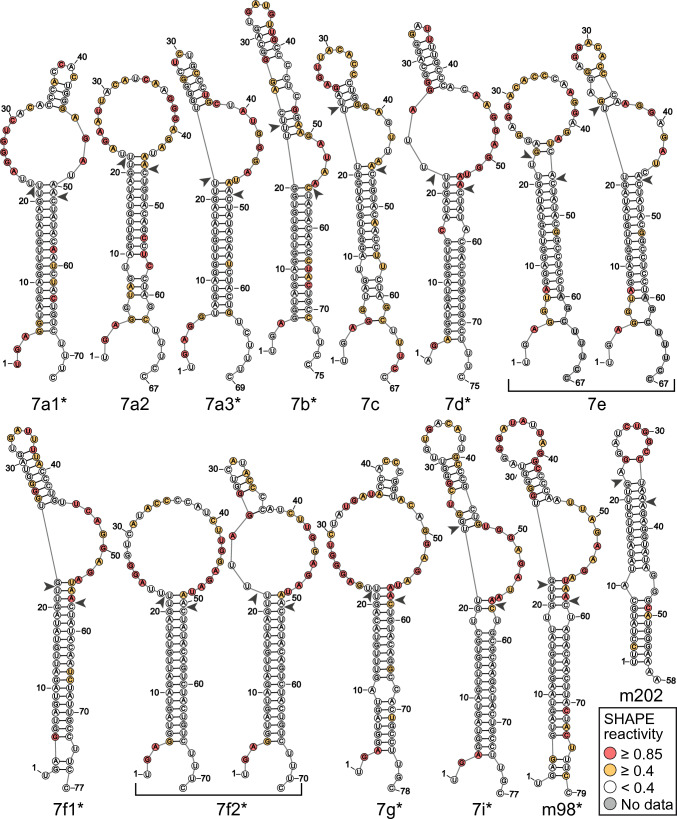
Secondary structures of the lowest energy conformer(s) for pre-let-7 RNAs determined by SHAPE analysis. The structures shown represent the lowest energy conformation(s), using a threshold of maximum energy difference with the lowest energy structure of 10% (Δ*E*_max_ = 10%; see Supplementary Table S2). The normalized 1M7 reactivity of each nucleotide is color-coded as per the SHAPE reactivity key. Dicer cleavage sites according to miRBase are indicated with an arrowhead and Group II pre-let-7 are annotated with a star

To systematically determine the secondary structures of all pre-let-7 miRNAs, SHAPE probing was performed using the fast-acting reagent 1M7, and secondary structures were determined using the RNAstructure software [58] (Fig. 1). A single structure defines the 10% lowest-energy conformation(s) of most let-7 members, except for 7e and 7f2, which are defined by two energetically similar conformations that likely co-exist in solution (Supplementary Table S2). When considering the 50% lowest-energy conformations, a few other pre-let-7 members are defined by more than a single conformation, namely 7f1, 7i, and m98 (Supplementary Fig. S1). The lowest-energy structures derived from SHAPE data are generally different from those reported in miRBase [49] or predicted by mFold based on nearest neighbor thermodynamic rules (Supplementary Fig. S2) [51]. We also found some differences with secondary structures derived from chemical probing data for 7a1, 7c, 7f1, 7g, and m98 that may result from variations in experimental conditions [19, 39], although our 7g structure is in good agreement with dsRNA cleavage data [38].

These SHAPE-derived structures are in good agreement with the experimental SHAPE reactivities, with > 95% of highly reactive bases not being involved in canonical base-pairing or residing adjacent to helices, termini or bulges/loops. Reactive nucleotides in paired region are often associated with G–U base pairs and/or base-pair stacking that is less energetically favorable, as previously observed [63]. Non-reactive nucleotides in unpaired regions could be due to base stacking. Alternatively, it may suggest that the reactivity is not fully compatible with a single structure but rather is consistent with an ensemble of structures, as observed for the 10% lowest-energy conformations of 7e and 7f2 (Fig. 1) and the 50% lowest-energy conformations of 7f1, 7i, and m98 (Supplementary Fig. S1). Two types of apical loops are observed for the pre-let-7 RNAs in Fig. 1. In the first set, the pre-miRNAs form a large loop, either an internal loop (7a1, 7d, 7f2, and 7 g) or a terminal loop (7a2, 7e, 7f2, and m202). In the second set, the structures contain a large 3′ bulge near the cleavage site (7a3, 7b, 7c, 7e, 7f1, 7i, and m98). For all pre-let-7 members, except 7e, the 5p cleavage site is located exactly at the junction between the paired region and the large loop/bulge region or 1–2-nt away. For the latter cases, only minor conformational changes would be required to bring the 5p cleavage site in a dicing-compatible conformation, as found in recent cryo-EM structures of human Dicer in the dicing state [19, 21]. In contrast to current conception, SHAPE results indicate that several residues at the 5′/3′ ends of all pre-let-7 are mostly unpaired and flexible, except for m202. Thus, the SHAPE results are generally consistent with the view that pre-let-7 RNAs are best described by a dynamic ensemble of conformations, with each pre-let-7 having its own structural characteristics.

### Binding of let-7 pre-miRNAs to Dicer

For in vitro binding and kinetic studies of Dicer with precursors of the let-7 family, we used pre-let-7 substrates carefully prepared to replicate the cleavage products generated by Drosha (Fig. 1 and Supplementary Fig. S3a–c). Those RNAs were 5′-^32^P-end-labeled to enhance the sensitivity, precision, and quantitative aspects of our analyses while ensuring that the chemical structure of the 5′- and 3′-ends corresponds exactly to those of natural pre-let-7 substrates. This labeling approach is highly suitable for these studies given that it allows the observation 5′-cleavage products and that the let-7 miRNAs mature primarily from the 5p arm.

**Fig. 2:**
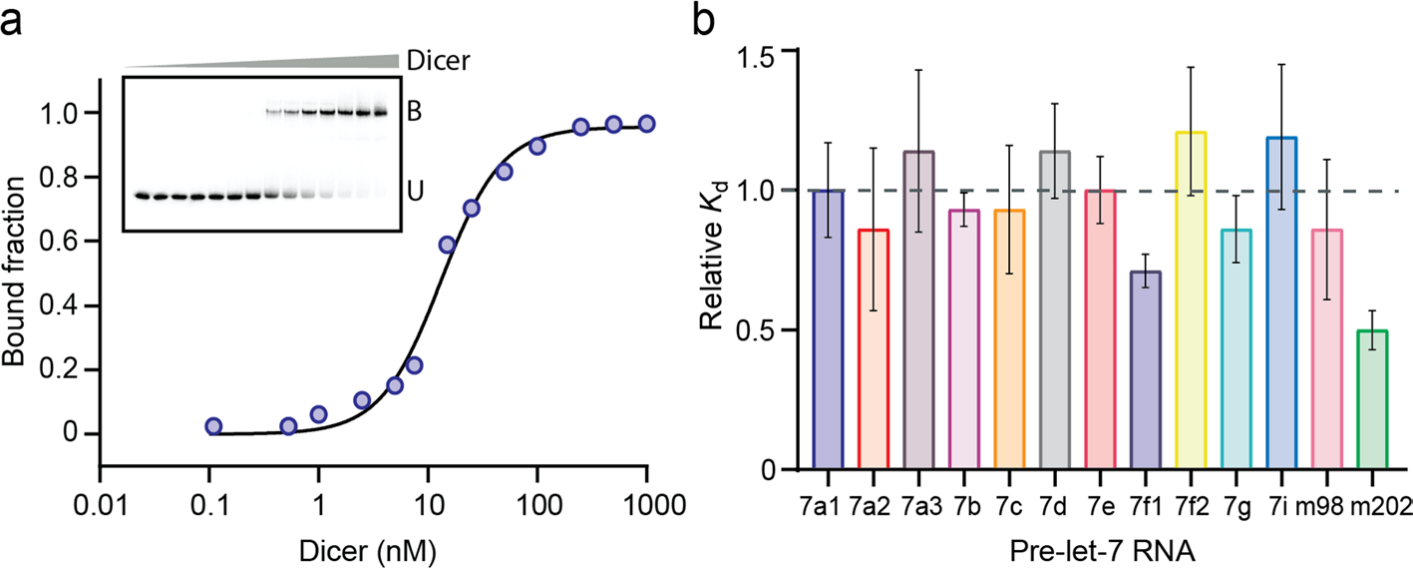
Binding studies of Dicer with pre-let-7 RNAs. **a** Typical EMSA performed with 5′-^32^P-labeled pre-let-7a-1 and increasing concentrations of ciDicer. The bound (B) and unbound (U) RNA fractions were analyzed on a 4–15% gradient native PAGE. The bound fractions were plotted against Dicer concentrations and the data were fitted to the Hill equation to obtain the dissociation constant (*K*_d_ = 12 nM). **b** Relative *K*_d_ values for all thirteen pre-let-7 were normalized using pre-let-7a-1 as the reference. The *K*_d_ values were obtained with standard deviation (shown by the error bars) from at least three independent experiments

For binding studies, we performed electrophoretic mobility shift assays using ^32^P 5′-end-labeled RNAs and catalytically inactive Dicer (ciDicer). Apparent dissociation constants (*K*_d_) were determined for all thirteen pre-let-7 members as shown for 7a1 (Fig. 2a). To allow a fair comparison between values obtained for different pre-let-7 members, experiments were performed under identical conditions using the same preparation of Dicer for all RNAs. We found that the *K*_d_ values for all thirteen pre-let-7 members range between 7 and 17 nM (Table 1), which is in good general agreement with previous studies of Dicer binding to 7a1 under similar conditions [29, 64]. A surprisingly small range (~ twofold) of *K*_d_ values were observed for the thirteen pre-let-7 members (Fig. 2b). Pre-miR-202 with its smaller apical loop (14-nt) has the lowest *K*_d_ value (7 nM), twofold smaller than the average of the twelve other members (13.7 ± 2.3 nM). Overall, it appears that the sequence and structural differences identified in the pre-let-7 RNAs (Fig. 1 and Supplementary Table S1), including the type of 3′-overhang (Group I versus Group II), do not affect Dicer binding.

**Table 1:** Summary of Dicer binding and kinetic constants for the pre-let-7 family

Pre-let-7 substrate	*K*_d_ (nM)	*k*_obs_ (min^−1^)	*K*_M_ (µM)	*k*_cat_ (min^−1^)	*k*_cat_/*K*_M_ (min^−1^ µM^−1)^
7a1^a^	14 ± 3	0.17 ± 0.03	0.93 ± 0.09	6.90 ± 0.20	7.42 ± 1.10
7a2	12 ± 5	0.14 ± 0.01	0.46 ± 0.18	1.40 ± 0.40	3.04 ± 1.50
7a3^a^	16 ± 5	0.153 ± 0.001	0.90 ± 0.06	4.60 ± 0.10	5.11 ± 0.40
7b^a^	13 ± 1	0.16 ± 0.03	0.11 ± 0.01	1.80 ± 0.04	16.36 ± 2.6
7c	13 ± 4	0.26 ± 0.03	0.27 ± 0.03	3.00 ± 0.10	11.11 ± 1.2
7d^a^	16 ± 3	0.14 ± 0.04	1.13 ± 0.11	17.2 ± 0.6	15.22 ± 1.5
7e	12 ± 2	0.22 ± 0.01	0.13 ± 0.01	1.60 ± 0.10	12.31 ± 1.0
7f1^a^	10 ± 1	0.120 ± 0.003	1.20 ± 0.13	8.70 ± 0.60	7.25 ± 0.90
7f2^a^	17 ± 4	0.17 ± 0.01	0.80 ± 0.08	8.40 ± 0.40	10.50 ± 1.1
7g^a^	12 ± 2	0.26 ± 0.03	0.24 ± 0.03	1.90 ± 0.60	7.92 ± 2.29
7i^a^	17 ± 5	0.18 ± 0.02	1.19 ± 0.16	3.20 ± 0.50	2.69 ± 0.50
m98^a^	12 ± 7	0.13 ± 0.01	0.36 ± 0.06	2.10 ± 0.20	5.83 ± 1.10
m202	7 ± 2	0.44 ± 0.02	1.83 ± 0.33	26.6 ± 1.8	14.53 ± 3.6

^a^Group II pre-let-7 RNAs

### Single-turnover kinetic studies for pre-let-7 cleavage by Dicer

**Fig. 3:**
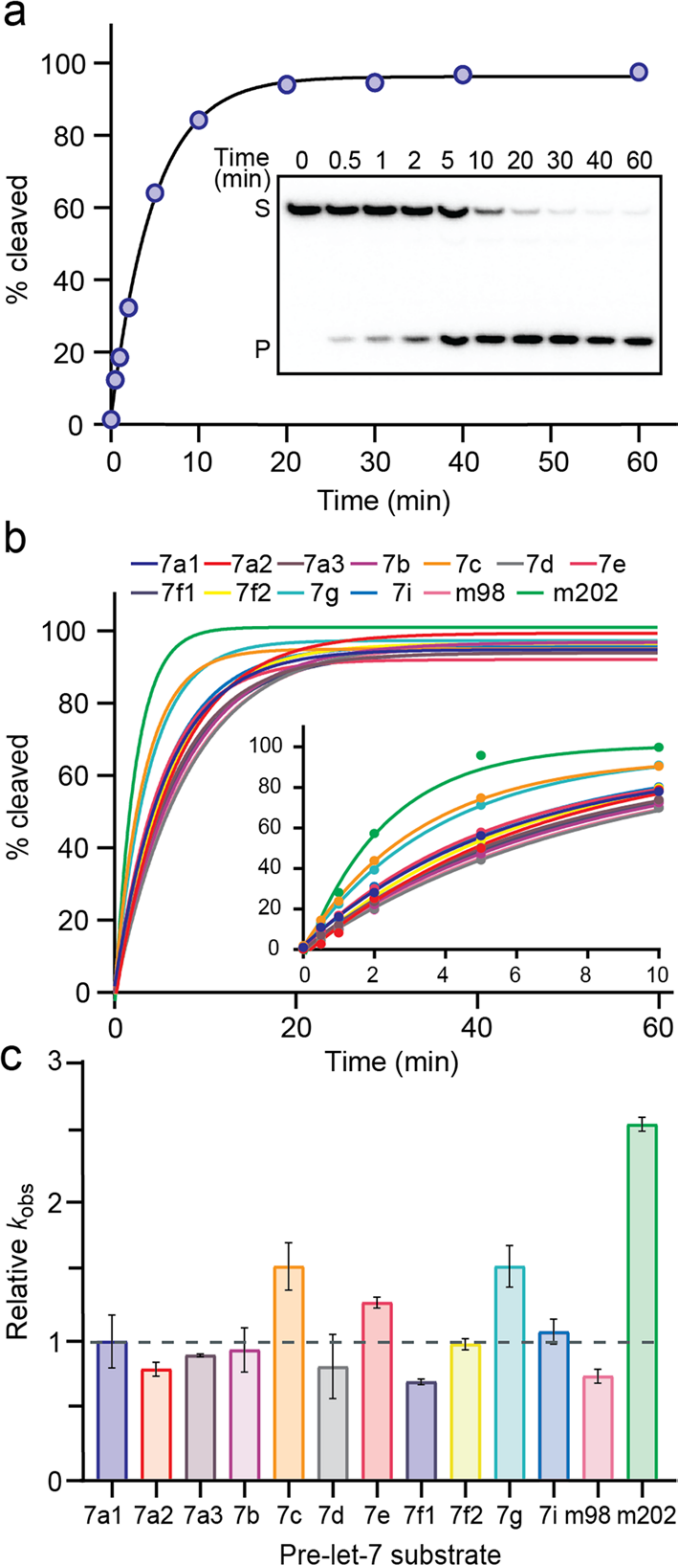
Single-turnover kinetics of pre-let-7 RNA cleavage by Dicer **a** Typical cleavage assay carried with 0.1 nM 5′-[^32^P]-labeled pre-let-7a-1 and 5 nM Dicer. The denaturing gel shows the relative quantity of substrate (*S*) and 5′-product (*P*) of aliquots taken at different time points (0–60 min). The exponential fit of this single-turnover kinetic data yields the values of *k*_obs_ (0.21 min^−1^) and maximum percentage of cleavage (96.2%) with an *R*^*2*^ value of 0.999. **b** Comparison of exponential fits of single-turnover kinetic data for the thirteen pre-let-7 showing the full time course and an insert with the early time points. **c** Relative *k*_obs_ values for all thirteen pre-let-7 using pre-let-7a-1 as the reference. In (**b**) and (**c**), the fits and *k*_obs_ values with standard deviation (shown by the error bars) were obtained from at least three independent experiments

To compare the cleavage activity of Dicer for different pre-let-7 members, single-turnover kinetics were performed with a 50-fold excess concentration of enzyme (5 nM) over the substrate (0.1 nM). The integrity and thermal stability of the Dicer protein used for our kinetic studies was first validated by SDS-PAGE before and after incubation at 37 °C for 30 min (Supplementary Fig. S3d). The observed rate constant (*k*_obs_) values were derived for all thirteen pre-let-7 substrates as shown for 7a1 (Fig. 3a). Cleavage data for all pre-let-7 fit well the single-exponential function describing pseudo-first-order kinetics and reaching a cleavage saturation of 93% to 98% (Fig. 3b). We found that the observed rate constant (*k*_obs_) values range between 0.12 and 0.44 min^−1^. (Table 1). These results indicate that Dicer cleaves these RNAs with very similar rates (within 3.4 folds) under the same single turnover conditions (Fig. 3c), with m202 having the fastest rate, 2.5-fold faster than the average of the twelve other members (0.175 ± 0.047 min^−1^). Moreover, cleavage by Dicer yields a single 22-nt let-7-5p product for all pre-let-7, except for 7e and 7f1 (Supplementary Fig. S4). In these cases, a minor 23-nt product is observed in addition to the main 22-nt let-7-5p product, with the percentage of the alternate product being 25% for 7e and 5% for 7f1. Overall, our single-turnover kinetic studies show that Dicer does not discriminate much between the pre-let-7 substrates, despite their sequence and structural differences.

### Catalytic efficiency of Dicer for pre-let-7 cleavage

**Fig. 4:**
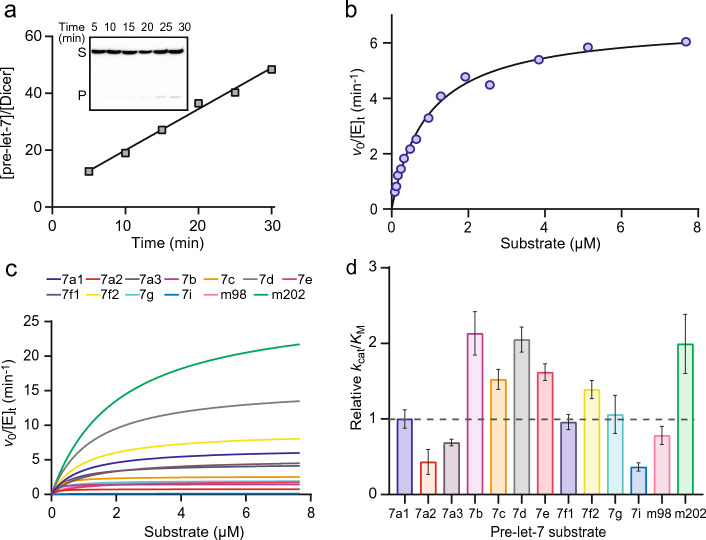
Steady-state kinetics of pre-let-7 RNA cleavage by Dicer. **a** Typical cleavage assay carried with 120 nM of pre-let-7a-1 and 0.25 nM Dicer. The denaturing gel shows the relative quantity of substrate (*S*) and 5′-product (*P*) of aliquots taken at different time points (5–30 min). The turnover frequency, expressed as *v*_0_/[*E*]_*t*_, was calculated by linear regression of the slope (0.82 ± 0.01 min^−1^). **b** Typical Michaelis–Menten plot for pre-let-7a-1 showing the dependence of *v*_0_/[*E*]_*t*_ on substrate concentration (0.08–7.68  μM). The Michaelis–Menten fit yields the values of *k*_cat_ (6.90 ± 0.20 min^−1^) and* K*_M_ (0.93 ± 0.09 µM^−1^ min^−1^) with an *R*^*2*^ value of 0.989. **c** Comparison of Michaelis–Menten fits for the thirteen pre-let-7. **d** Relative *k*_ca*t*_/*K*_M_ values for all thirteen pre-let-7 using pre-let-7a-1 as the reference and with the standard deviation shown by the error bars

Steady-state kinetics were performed to obtain information about the overall reaction pathway, including product release. The Michaelis–Menten parameters (*k*_cat_ and *K*_M_) and the specificity constant (*k*_cat_*/K*_M_) were determined for all thirteen pre-let-7 substrates. This was achieved by first determining the initial rate of the cleavage reaction at several different substrate concentrations and then fitting these values to the Michaelis–Menten equation as shown for 7a1 (Fig. 4a, b). For pre-let-7 substrates, the *K*_M_ values vary by 16 folds (0.11–1.8 μM) and the *k*_cat_ values by 19 folds (1.4–26.6 min^−1^); however, the *k*_cat_/*K*_M_ varies by only 6 folds (2.7–15.8 min^−1^ μM^−1^; Table 1; Fig. 4c, d). Two pre-let-7 substrates stand out with their high *k*_cat_ value (7d: 17.2 min^−1^ and m202: 26.6 min^−1^) and *K*_M_ values (7d: 1.13 μM and m202: 1.83 μM). Overall, the small difference in *k*_cat_/*K*_M_ values shows that Dicer’s catalytic efficiency is comparable for different pre-let-7 substrates, and thus, the enzyme has similar specificity for all pre-let-7 substrates, including those of the Group I and Group II families. Moreover, for all pre-let-7 substrates, the *K*_M_ value is greater than the *K*_d_ value (between 8.5-fold and 261-fold greater), indicating that in all cases catalysis occurs somewhat faster than substrate dissociation.

### Effect of 3′ mono-uridylation on pre-let-7 processing by Dicer

**Fig. 5:**
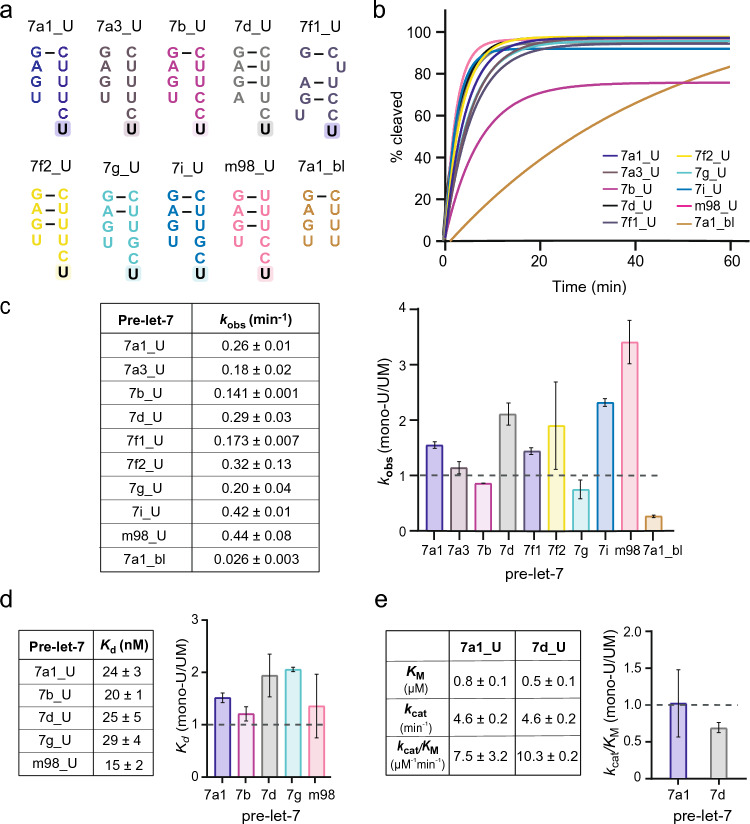
Effect of mono-uridylation on pre-let-7 cleavage and binding by Dicer. **a** Extremities of the mono-uridylated group II pre-let-7 construct with an extra uridine at the 3′-end (shaded). **b**, **c** Single turnover kinetics studies for all nine mono-uridylated group II pre-let-7 and a control blunt pre-let-7a-1 (7a1_bl). **b** Comparison of exponential fits for single-turnover kinetic data. **c** Reported average *k*_obs_ values and histogram showing relative *k*_obs_ values for all modified substrate (mono-U; mono-uridylated or blunt end) with respect to their unmodified (UM) counterparts. In (**b**) and (**c**), the fits and *k*_obs_ values with standard deviation (shown by the error bars) were obtained from at least three independent experiments. **d** Binding studies of selected mono-uridylated group II pre-let-7. The reported average *K*_d_ values were derived with standard deviation from at least three independent experiments. The histogram shows relative *K*_d_ values obtained for mono-uridylated substrate (mono-U) with respect to their unmodified (UM) counterparts. **e** Steady-state kinetics studies of selected mono-uridylated group II pre-let-7. The reported average steady-state kinetic values were derived with standard deviation from two independent experiments. The histogram shows relative *k*_cat_/*K*_M_ values obtained for selected mono-uridylated substrate (mono-U) with respect to their unmodified (UM) counterparts

In our investigations, Group I and Group II pre-let-7 RNAs bind Dicer with similar affinities and are cleaved by Dicer with similar rates (*k*_obs_ and *k*_cat_/*K*_M_). In contrast, a previous study shows that an immuno-purified Dicer could process mono-uridylated Group II substrates more efficiently than the unmodified substrate in vitro [41]. Thus, to directly test the effect of mono-uridylation using recombinant Dicer purified from human cells, Dicer binding and cleavage studies were performed with the 3′-mono-uridylated form of all nine Group II pre-let-7 substrates as well as with a blunt-end control (7a1_bl; Fig. 5a).

Dicer cleavage assays were first performed under single-turnover conditions and the exponential fits of the cleavage reactions were compared (Fig. 5b). We found that the cleavage for all mono-uridylated pre-let-7 substrates are rather similar, and they reach cleavage saturation above 90%, except for 7b_U (75%). The 7a1_bl control is cleaved slower than mono-uridylated pre-let-7 and does not reach cleavage saturation during the first 60 min of the reaction. Our results show that the mono-uridylated forms of pre-let-7 are cleaved with rates (*k*_obs_ values between 0.17 and 0.44 min^−1^; Fig. 5c) that are overall similar to those obtained for their unmodified form (0.12–0.26 min^−1^). When directly comparing *k*_obs_ values of individual mono-uridylated pre-let-7 with their unmodified counterpart, the most considerable difference is a 3.6-fold increase for m98. Otherwise, we observed a twofold increase for 7d_U, 7f2_U, and 7i_U and values within 1.5-fold for all other substrates. In contrast, the *k*_obs_ value for the blunt control (7a1_bl) is sevenfold lower than for the unmodified 7a1.

Dicer binding studies with five 3′-mono-uridylated Group II pre-let-7 substrates (7a1, 7b, 7d, 7g, and m98) show that Dicer binds these mono-uridylated pre-miRNA (*K*_d_ of 15–24 nM) with similar affinities (Fig. 5d, left panel). When compared individually to their unmodified counterparts (*K*_d_ of 7–17 nM; Table 1), the mono-uridylated pre-let-7 bind with slightly lower affinity, within twofold of less (Fig. 5d, right panel). Moreover, steady-state kinetics for mono-uridylated 7a1 and 7d reveal that the *k*_cat_/*K*_M_ values are very similar between the mono-uridylated (between 7.5 and 10.3 μM^−1^ min^−1^) and unmodified RNAs (between 7.4 and 15.2 μM^−1^ min^−1^; Table 1), with individual differences being less than 1.5-fold (Fig. 5e). Overall, little differences were observed in the binding and cleavage by Dicer between mono-uridylated and unmodified pre-let-7 substrates.

Since it was previously shown that an immuno-purified Dicer can process mono-uridylated Group II substrates more efficiently than unmodified substrates [41], a cofactor may be important to allow Dicer to discriminate between these two substrates. Thus, we conducted cleavage studies of 7a1 in the presence of the well-known Dicer cofactor TRBP [65–67]. Under single-turnover conditions, addition of TRBP does not have a significant effect on the observed cleavage rate of both 7a1 (0.17 ± 0.01 min^−1^) and 7a1_U (0.31 ± 0.02 min^−1^) compared to their respective rates without TRBP (0.21 ± 0.01 min^−1^ and 0.26 ± 0.01 min^−1^) (Fig. 5e and Supplementary Fig. S5a). Thus, as previously observed, TRBP does not affect the cleavage rate of 7a1 under single-turnover conditions [64], and, interestingly, we make the same observation for 7a1_U. In contrast, under multiple turnover conditions, the presence of TRBP enhances the cleavage efficiency of 7a1, as previously observed [64] (Supplementary Fig. S5b). Nevertheless, the multiple-turnover cleavage profiles for 7a1 and 7a1_U in the presence of TRBP are nearly identical. Thus, Dicer cleaves mono-uridylated and unmodified pre-let-7a1 at similar rates both in the absence and presence of TRBP and regardless of the kinetic conditions (single or multiple turnover).

### Effect of oligo-uridylation on pre-let-7 processing by Dicer

**Fig. 6:**
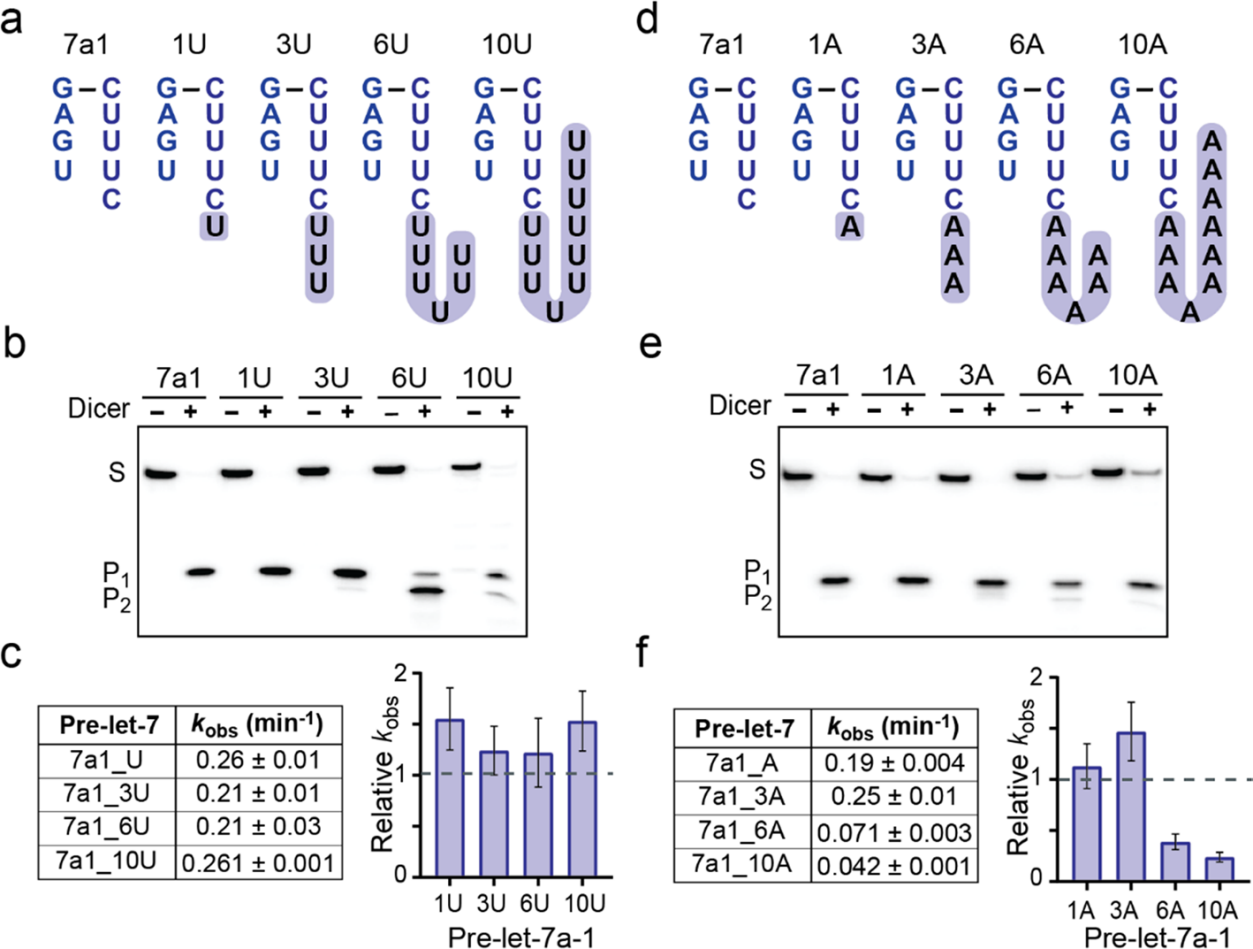
Effect of oligo-uridylation and oligo-adenylation on pre-let-7 cleavage by Dicer. **a**, **d** End structures of pre-let-7a-1 RNAs with extra uridines or extra adenines at the 3′-end (shaded). **b**, **e** Dicer cleavage products for unmodified (7a1) and modified pre-let-7a-1 RNAs (**b**: uridylated and **e**: adenylated). The cleavage assays were carried under single-turnover conditions (0.1 nM substrate and 5 nM Dicer) for 15 min. The standard 22-nt miRNA product (*P*_1_) and a shorter alternate product (*P*_2_) were separated from the substrate (*S*) by denaturing gel. **c**, **f** Reported average *k*_obs_ values and histogram showing relative *k*_obs_ values for modified pre-let-7a-1 RNAs compared to the unmodified 7a1 (0.17 min^−1^) (**c**: uridylated and **f**: adenylated). In (**c**) and (**f)**, the *k*_obs_ values with standard deviation (shown by the error bars) were obtained from at least three independent experiments

To investigate the effect of oligo-uridylation on Dicer processing, pre-let-7a-1 RNAs containing additional uridines at their 3′-end were prepared (3U, 6U and 10U; Fig. 6a), and their cleavage by Dicer was assayed from a 20-min single-turnover reaction. Cleavage by Dicer produced the same 5p-miRNA product (*P*_1_) for 7a1 and its uridylated counterparts (1U, 3U, 6U and 10U; Fig. 6b). However, an additional product (P_2_) was observed for the oligo-uridylated substrates (3U, 6U, and 10U), with *P*_2_ being less than 5% of the total products for 3U and 10U, but the most abundant one for 6U. Sequencing gel analysis was used to confirm that *P*_1_ is the expected 22-nt product and to define *P*_2_ as being 17-nt long (Supplementary Fig. S6). A closer examination of the 6U cleavage time course reveals that the appearance of the two products is intertwined (Supplementary Fig. S7). While *P*_1_ formation dominates at shorter time points to peak to 40% of total products at 10 min and then decline to 20% at 60 min, formation of *P*_2_ is slower at first but essentially follows an exponential model, reaching 80% of total products at 60 min. Thus, it appears that formation of the 17-nt *P*_2_ product results at least in part from a secondary cleavage event, whereby a 5-nt fragment at the 3′-end of *P*_1_ is cleaved off. From single-turnover kinetic studies, *k*_obs_ values of 0.21 to 0.26 min^−1^ were obtained for cleavage of 3U, 6U, and 10U (Fig. 6c, left panel). Surprisingly, the *k*_obs_ values for the oligo-uridylated RNAs are very similar to those of the unmodified 7a1 substrates (Fig. 6c, right panel), the largest difference observed being 1.5-folds. Thus, even though Dicer cleavage of oligo-uridylated pre-let-7a-1 substrates can yield a shorter additional product, 3′-uridylation does not affect the rate of the single-turnover cleavage reaction.

### Effect of mono- and oligo-adenylation on pre-let-7 processing by Dicer

Terminal nucleotidyl transferases (Tents) such as Tent1 (also known as Tut1) and Tent2, can also mono-adenylate the 3′ end of pre-miRNAs to stabilize miRNAs [42], and this modification may affect Dicer activity. To test this possibility, we performed Dicer processing with mono-adenylated pre-let-7a-1 (1A; Fig. 6d). Under single-turnover conditions, Dicer was found to cleave the mono-adenylated substrate with a rate (*k*_obs_ = 0.19 min^−1^) that is very similar to that of its unmodified counterpart (*k*_obs_ = 0.17 min^−1^; Table 1 and Fig. 6e, f).

Although oligo-adenylation of pre-miRNAs by human Tents has not been observed in vitro, it may occur in vivo through the assistance of RNA binding proteins [68]. To study the effect of oligo-adenylation on Dicer processing, pre-let-7a-1 RNAs were prepared that contain additional adenines at their 3′-end (3A, 6A and 10A; Fig. 6d), and their cleavage by Dicer was first assayed from a 20-min single-turnover reaction. Cleavage by Dicer produced the same 5p-miRNA product (*P*_1_) for 7a1 and its adenylated counterparts (1A, 3A, 6A, and 10A), with only minor amounts (< 5%) of an additional 17-nt product for the oligo-adenylated substrates (Fig. 6e and Supplementary Fig. S6). However, a greater proportion of the uncleaved substrate is observed for 6A and 10A compared to 7a-1, 1A, and 3A. In agreement with this observation, *k*_obs_ values for cleavage of 6A and 10A are reduced by 2.6- and 4.3-folds, respectively, compared to the unmodified 7a1 (Fig. 6f), whereas the rates of 1A and 3A are within 1.5 folds of 7a1. Thus, although a small adenylate tail of 1–3 residues does not affect the rate of cleavage by Dicer, a longer oligo-adenylate tail (6–10 residues) reduces to some extent the rate of pre-let-7 cleavage by Dicer.

## Discussion

In this study, we first defined the lowest-energy secondary structure(s) of all pre-let-7 members using SHAPE probing, highlighting their individual characteristics and inherent dynamics. Surprisingly, we found that the 5′/3′ ends of most pre-let-7 are flexible and do not form stable base pairs. Given that less stable pre-miRNA ends are known to be readily captured by Dicer and cleaved from the 5′-end (5′ counting rule) [25, 27], pre-let-7 RNAs probably use a similar mechanism. Upon Dicer binding, the 5′/3′ ends may be stabilized via formation of additional base pairs, leaving a 1-nt or 2-nt 3′-overhang, although it is not clear if formation of these base pairs is required for cleavage by Dicer. Nevertheless, a flexible 5′/3′-end may help recognition by other RNA-binding proteins, such as TUTases, that regulate let-7 maturation and stability.

Detailed thermodynamic and kinetic investigations with in vitro purified Dicer revealed that despite structural differences among the thirteen pre-let-7 RNAs, Dicer does not discriminate much between these substrates. The most notable disparities among the results were observed for miR-202, which displays somewhat higher affinity for Dicer and faster cleavage rates, possibly due to some of its unique features like its smaller apical loop [28, 29]. The poor discrimination of Dicer for different pre-let-7 substrates aligns with prior research demonstrating that human Dicer tolerates significant structural variations in its natural substrates [28, 29, 33]. Moreover, given that the *K*_M_ value is much greater than the *K*_d_ value for all pre-let-7 substrates, these are considered “sticky” substrates for Dicer, which means that nearly every instance of substrate binding to Dicer results in cleavage. This enzymatic behavior is generally associated with broad substrate specificity [69], as observed here for Dicer and its pre-let-7 substrates. However, these observations do not align with previous reports showing that pre-miRNAs with a 1-nt 3′-overhang are cleaved less efficiently by Dicer [40–42].

Investigations of the mono-uridylated form of Group II pre-let-7 also support this change of paradigm, revealing that the reported thermodynamic and kinetic parameters are hardly affected by the extra uridine at their 3′-end. Similar observations were made based on kinetic studies of pre-let-7a1 in the presence of TRBP. Thus, we conclude that mono-uridylation does not significantly affect Group II pre-let-7 cleavage by Dicer. Mono-uridylation may promote let-7 maturation and stability, but not by directly enhancing Dicer cleavage activity. Instead, mono-uridylation may prevent binding of protein factors that induce pre-let-7 degradation or promote binding of proteins that protect pre-let-7 from degradation. In summary, our in vitro studies on Dicer binding and cleavage indicate that Dicer does not discriminate between the 1-nt and 2-nt 3′-overhang of the pre-let-7 substrates. These remarkable findings challenge our established understanding of the importance of a 2-nt 3′-overhang for efficient cleavage by Dicer.

Oligo-uridylation of pre-let-7 is known to promote degradation by the exonuclease Dis3L2 [43, 45], and oligo-uridylated pre-let-7 are often viewed as being poor substrates for Dicer [40, 48]. Surprisingly, we found that Dicer cleaved oligo-uridylated and unmodified 7a1 substrates with similar rates. However, we observed a second cleavage product for 7a-1_3U, 7a1_6U, and 7a1_10U. This result differs from previous observations where 7a1 with extra Us at the 3′-end yielded minor products of shorter sizes that varied in length according to number of added Us and were thought to originate from a 3′-counting model [27]. In contrast, our results indicate that this product is 17-nt long no matter the length of the 3′-oligo-uridylation tail and, thus, does not result from Dicer using a 3′ counting rule since this would entail that the product size depends on the number of extra residues at the 3′ end [27, 32], which is not the case. Moreover, analysis of the kinetic time course for 7a1_6U cleavage revealed a unique mechanism whereby Dicer cleaves the 22-nt 5p miRNA product to yield a secondary 17-nt product that becomes prominent as the reaction proceeds. A similar phenomenon has been reported previously in which human recombinant Dicer could cleave an RNA substrate with 5′-overhangs twice in vitro [70]. Further investigations are required to understand exactly why this secondary activity of Dicer is most important with the 7a1_6U substrate. One possible mechanism involves the normal production of the miRNA duplex that would then change its position with respect to the enzyme active site possibly due to changes in how the PAZ domain interacts with its 3′-end.

Although 3′-oligo-uridylation of 7a1 does not affect its cleavage rate by Dicer, 3′-oligo-adenylation lowers the cleavage rate by Dicer for 7a1_6A and 7a1_10A. It is puzzling that the nucleotide identity of the longer 3′-oligo tails differently affects the cleavage rate by Dicer since it is unlikely that these tails directly interact with Dicer. However, the longer 3′-oligo-adenylated tails (6A and 10A) may specifically affect the structural integrity of the RNA substrates and thereby inhibit their cleavage. Given that residues at the 5′/3′ ends of pre-let-7a1 are not stably paired, there is a possibility for these 3′-oligo-adenylated tails (but not for the 3′-oligo-uridylated tails) to base pair with the immediately upstream U-rich region (Fig. 6d), which may perturb the structural integrity of both the 5′- and 3′-ends and thereby hinder substrate recognition by the PAZ domain.

**Fig. 7:**
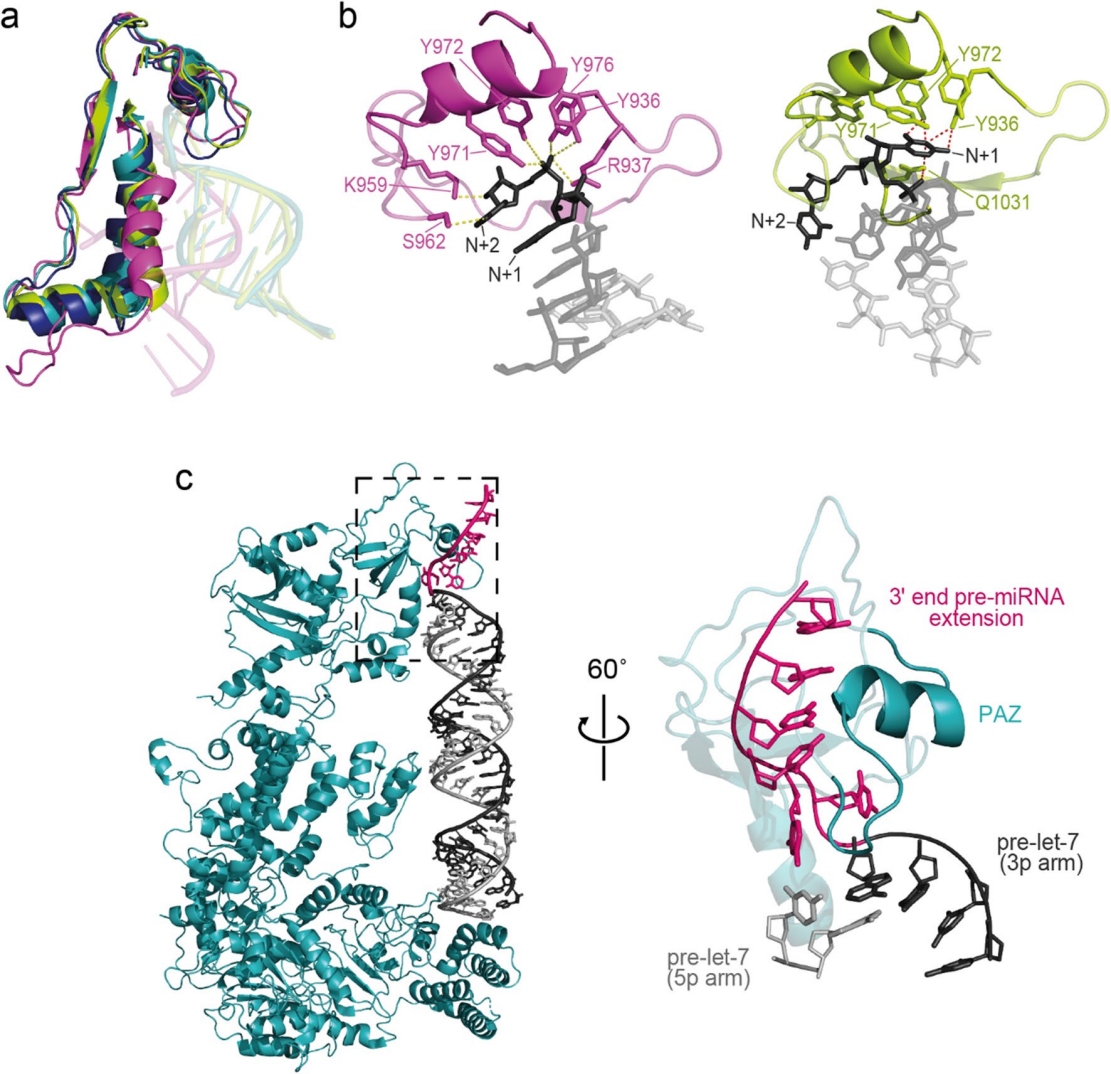
Structural understanding of the promiscuity of Dicer cleavage toward different substrates. **a** Alignment of cryo-EM structures of human Dicer free (PDB entry 5ZAK, dark blue) and in complex with pre-let-7a-1 variants [PDB entries 5ZAL (cyan; pre-dicing state with a full RNA stem) and 5ZAM (green-yellow; pre-dicing state with a partial RNA stem) and 7XW2 (pink; dicing state)] are shown [19, 21]. The platform-PAZ-connector (PPC) was aligned for all structures and the regions of interaction between the PAZ and RNA substrates are shown. **b** Stable interactions of pre-let-7a-1 variants with the 3′ binding pocket of the PAZ domain in selected structures of human Dicer. The uracil at the *N* + 2 position interacts with Tyr936, Arg937, Lys959, Ser962, Tyr971, and Tyr972 [21] in a structure of the dicing state (left panel; PDB entry 7XW2) [21], whereas the cytosine at the *N* + 1 position interacts with Tyr936, Tyr971, Tyr972, and Gln1031 in a structure of the pre-dicing state (right panel; PDB 5ZAL) [19]. **c** Cryo-EM model of human Dicer (blue) bound to a modified pre-let-7a-1 (cyan) containing an extension of 4 uridines at the 3′-end (a 6-nt 3′-overhang; magenta). This model was build from pdb entry 5ZAL [19]. The zoom-in view (right panel) shows the relevant region of the structure represented by a dotted box (left panel) and the absence of steric hindrance between the 3′ extension and the PAZ domain

This work reveals that most 3′-end modifications do not affect the rate of pre-let-7 cleavage by Dicer. We sought to understand if these results are consistent with our current structural understanding of human Dicer in which the platform-PAZ-connector (PPC) domain interacts with the 5′/3′-end of pre-miRNAs. A certain level of plasticity within the PAZ domain was previously observed from crystal structures of the human PPC domain in complex with different siRNAs (Supplementary Fig. S8a) [25] and cryo-EM structures of human Dicer in complex with different pre-let-7a-1 variants (Supplementary Fig. S8b, c) [19, 21]. However, the adaptability of the PAZ domain is more readily observed from structural rearrangements associated with the cleavage activity of human Dicer (Fig. 7a), first a minor change to bind the RNA (pre-dicing state) and then a major change to bring the bound RNA closer to the catalytic center to achieve a cleavage-competent state (dicing state) [19, 21]. This plasticity of the PAZ domain in different states is not unique to human Dicer, as it was also found in the cryo-EM structure of mouse Dicer in complex with pre-miR-15a (Supplementary Fig. S8d) [20]. These conformational changes within the PAZ domain likely reflect its inherent flexibility and support the ability of Dicer to cleave with similar rates pre-let-7 substrates with different 3′-ends.

Recent cryo-EM structures of human Dicer further illustrate the adaptability of the PAZ domain for recognition of the 2-nt 3′-overhang. Whereas the structure of a Dicer/pre-let-7 complex in the dicing state [21] provides evidence for the interaction of the PAZ domain with the sugar-phosphate backbone of the terminal nucleotide (*N* + 2) (Fig. 7b, left panel), the structure of a Dicer-TRBP-pre-let-7 complex in a pre-dicing state [19] shows PAZ interacting exclusively with the penultimate residue (*N* + 1) of the 2-nt 3′-overhang (Fig. 7b, right panel). These observations are fully consistent with results from kinetic studies of cleavage by Dicer showing similar rates for Group I and Group II pre-let-7 substrates as well as for mono-uridylated Group II substrates. More generally, our binding and kinetic data suggest that as long as an interaction between the PAZ domain and either the *N* + 1 or *N* + 2 nt of pre-let-7 can occur, the 3′-end extension does not affect the cleavage rate by Dicer. Furthermore, to verify that 3′-end oligo-uridylation of 7a1 is also compatible with the structure of the human Dicer-TRBP-pre-let-7 complex, a 6-nt 3′-end extension was modeled as an A-form helix onto this complex (Fig. 7c). Interestingly, no steric hindrance was found between the extended 3′-ends and the nearby PPC of Dicer, in agreement with our single-turnover kinetic results of pre-let-7 cleavage by Dicer, where the observed cleavage rates are similar for the unmodified and 3′-oligo-uridylated substrates. Future high-resolution structural studies of human Dicer in complex with different substrates are required to fully support our results highlighting the remarkable substrate promiscuity of Dicer.

In conclusion, we found that human pre-let-7 RNAs adopt diverse secondary structures in solution, and most of them contain several mostly unpaired and flexible residues in the apical loop, dsRNA region, and at the 5′/3′ ends. We demonstrated that despite these structural discrepancies, Dicer displays promiscuity in its binding and cleavage activity toward these pre-let-7 substrates, with some slight preference for the shorter m202 substrate. In contrast with the current paradigm, mono-uridylation has little effect on Dicer binding constants and cleavage rates of Group II pre-let-7 RNAs, indicating that Dicer displays little preference for 2-nt over 1-nt 3′-overhangs. Moreover, 3′-oligo-uridylation of pre-let-7a-1 was found not to affect its cleavage rate by Dicer. Yet, 3′-oligo-uridylation may yield an additional 5p miRNA product of 17-nt. These in vitro investigations of Dicer binding and cleavage of let-7 precursors allow us to update current concepts related to Dicer activity toward different substrates, accentuating the broad specificity of this key enzyme in RNA silencing. This broad specificity is likely important for cleavage of a wide variety of other known substrates, such as dsRNAs, Line1 retrotransposons, mRNAs, snoRNAs, tRNAs, and harmful R-loops [16, 31, 71, 72].

## Supplementary Information

Below is the link to the electronic supplementary material.

Supplementary file1 (PDF 1144 KB)

## Data Availability

The datasets generated during and/or analyzed during the current study are not publicly available due to the fact that there is no depository for this type of data but additional data are included as electronic supplementary material and are available from the corresponding author on reasonable request.
