# Correction: Substrate promiscuity of Dicer toward precursors of the let-7 family and their 3′-end modifications

**DOI:** 10.1007/s00018-025-05795-6

**Published:** 2025-06-27

**Authors:** Gunjan Dadhwal, Hebatallah Samy, Jonathan Bouvette, Fatima El-Azzouzi, Pierre Dagenais, Pascale Legault

**Affiliations:** 1 Département de biochimie et médecine moléculaire, Université de Montréal, Downtown Station, Box 6128, Montreal, QC H3C 3J7 Canada; 2 Molecular Biology Department, Guyot Hall, Princeton University, Princeton, NJ 08544 USA; 3 Biochemistry Department, Wake Forest Biotech Place, 575 Patterson Avenue, Winston-Salem, NC 27101 USA

**Correction: Cellular and Molecular Life Sciences (2024) 81:53**

10.1007/s00018-023-05090-2

In this article the author’s name Hebatallah Samy Saad was incorrectly written as Hebatallah Samy.

The original article has been corrected.

