# Supplementary material for: Substrate promiscuity of Dicer toward precursors of the let-7 family and their 3′-end modifications
Source: Cell Mol Life Sci. 2024 Jan 23;81(1):53. doi: 10.1007/s00018-023-05090-2 (PMC10806991; doi:10.1007/s00018-023-05090-2)
Supplement: Supplementary file 1 — Supplementary file1 (PDF 1144 KB) [file 18_2023_5090_MOESM1_ESM.pdf]

Supplementary Information

Supplementary Tables

Table S1. Structural features of pre-let-7 RNAs

Pre-let-7 RNA	Size (nt)	Size of apical loop (nt)	Group[a]	Unpaired nt on 5p arm	Unpaired nt on 3p arm	Extra unpaired nt at 3'-end	Cleavage site on 5p-arm	Cleavage site on 3p-arm
**7a1***	72	27	II	1-3	69-72	1	UU-UU	AA-CU
**7a2**	67	21	I	1-3, 5, 9	57, 61, 63-67	2	UU-UA	AA-CU
**7a3***	69	24	II	1-5	64-69	1	UU-UG	AA-CU
**7b***	75	30	II	1-3	72-75	1	UU-UC	AA-CU
**7c**	67	20	I	1-3, 5, 9	57, 61, 63-67	2	UU-UA	AA-CU
**7d***	75	30	II	1, 2, 16	59, 73-75	1	UU-UU	AA-CU
**7e**	67	22	I	1-3, 5	61, 63-67	2	UU-GA	CA-CU
**7f1***	77	34	II	1	74, 77	0 or 1	UU-GU	AA-CU
**7f2***	71	26	II	1-3	68-71	1	UU-UU	AA-CU
**7g***	78	33	II	1, 2, 10	68, 76-78	1	UU-UG	AA-CU
**7i***	77	32	II	1, 2, 19	68, 75-77	1	UU-GG	AA-CU
**m98***	79	34	II	1, 19	60, 75, 78, 79	1	UU-GU	AA-CU
**m202**	58	14	I	10	46, 47, 57, 58	2	UG-AG	AA-AG

* Group II pre-let-7 RNAs

[a] Group I pre-let-7 have a 2-nt 3'-overhang and Group II pre-let-7 have a 1-nt 3'-overhang [1].

Table S2. RNA structure free energies of lowest-energy conformer(s) (ΔE_{max} = 10%) for pre-let-7 RNAs determined by SHAPE.

Pre-let-7 RNA	Energy (kcal/mol)
**Pre-let-7a-1**	-59.8
**Pre-let-7a-2**	-40.6
**Pre-let-7a-3**	-63.9
**Pre-let-7b**	-66.5
**Pre-let-7c**	-43.0
**Pre-let-7d**	-76.7
**Pre-let-7e (conformation 1)**	-43.0
**Pre-let-7e (conformation 2)**	-42.9
**Pre-let-7f-1**	-75.7
**Pre-let-7f-2 (conformation 1)**	-64.3
**Pre-let-7f-2 (conformation 2)**	-63.7
**Pre-let-7g**	-51.4
**Pre-let-7i**	-73.5
**Pre-miR-98**	-59.4
**Pre-miR-202**	-59.8

Supplementary Figures

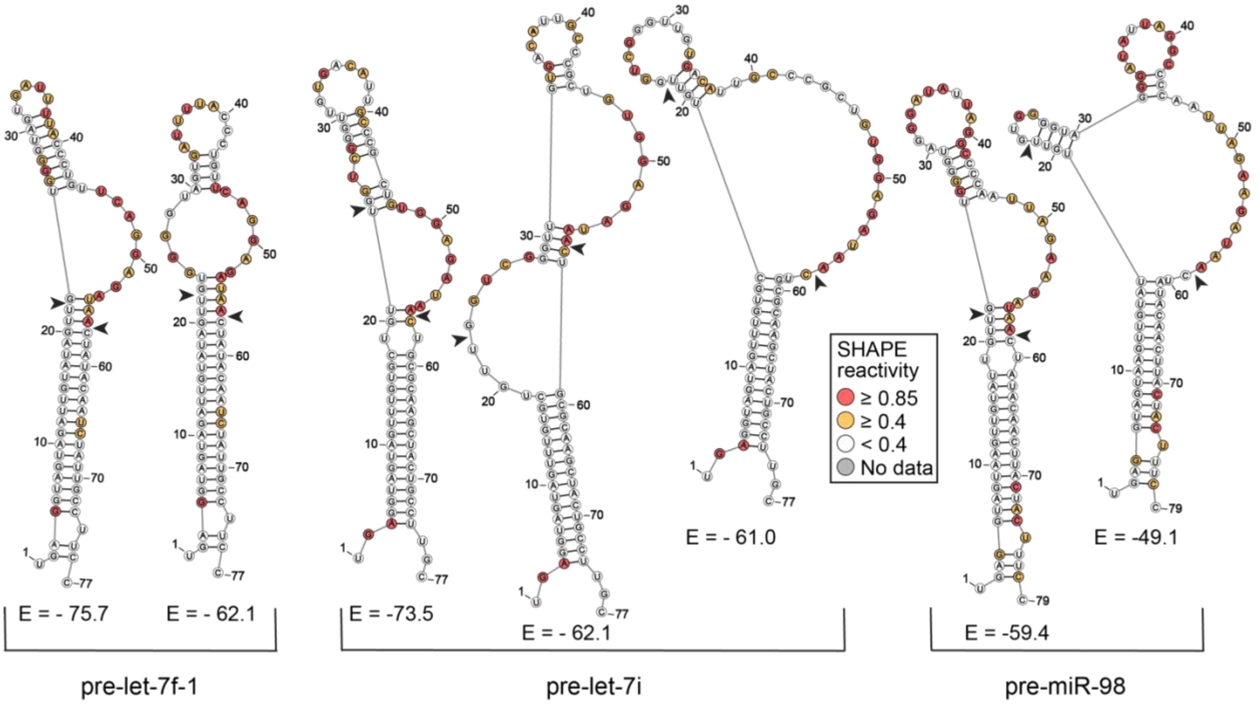

Fig. S1 Secondary structures of the lowest-energy conformers for selected pre-let-7 RNAs determined by SHAPE. The structures shown represent the lowest energy conformation(s), using a threshold of maximum energy difference with the lowest energy structure of 50% (ΔE_{max} = 50%). The energies of the structure provided by the RNAstructure software are given in kcal/mol. The normalized 1M7 reactivity of each nucleotide is color-coded as per the SHAPE reactivity key. Dicer cleavage sites are indicated with an arrowhead.

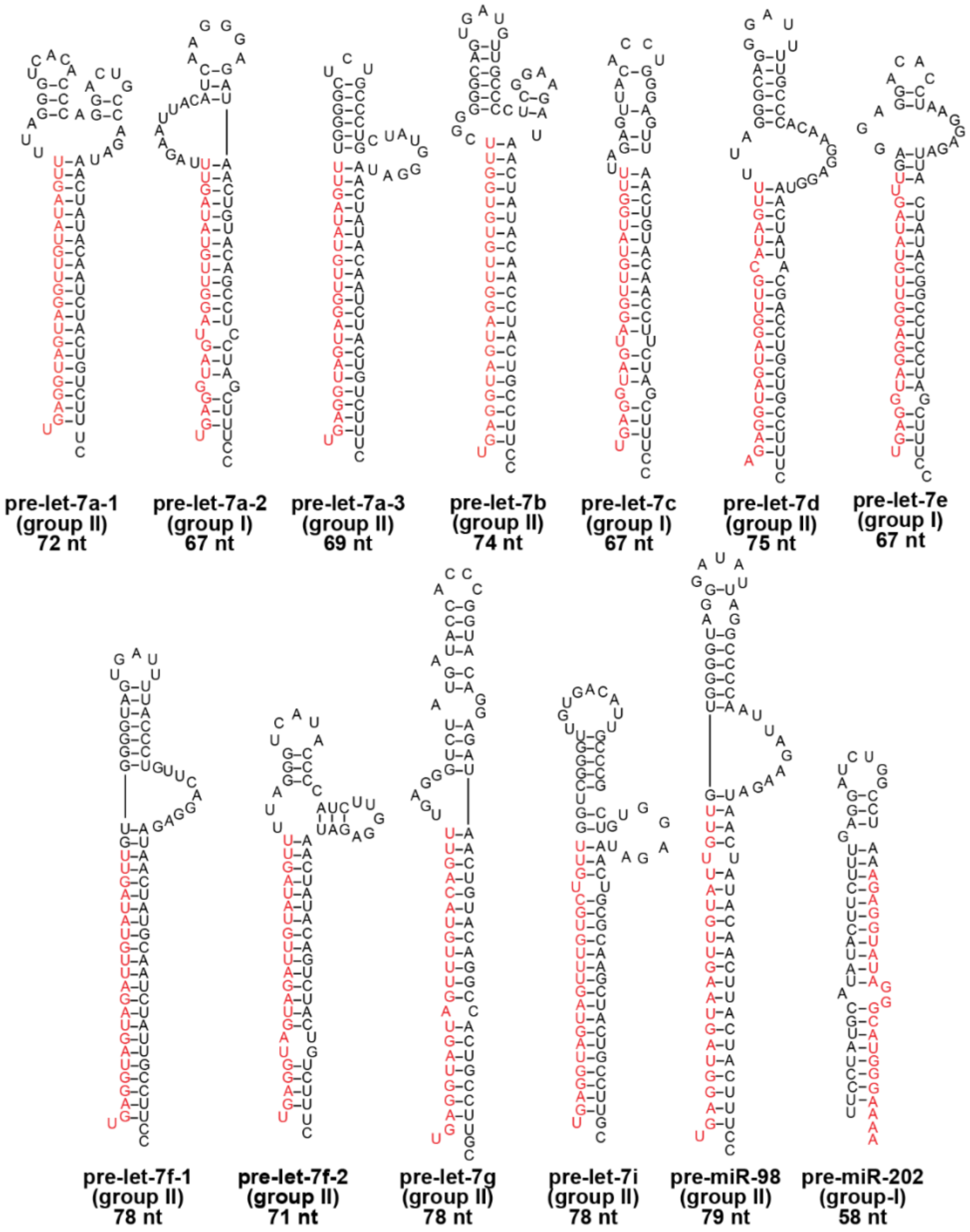

Fig. S2 Secondary structure of all pre-let-7 RNAs predicted with the mfold software [2].

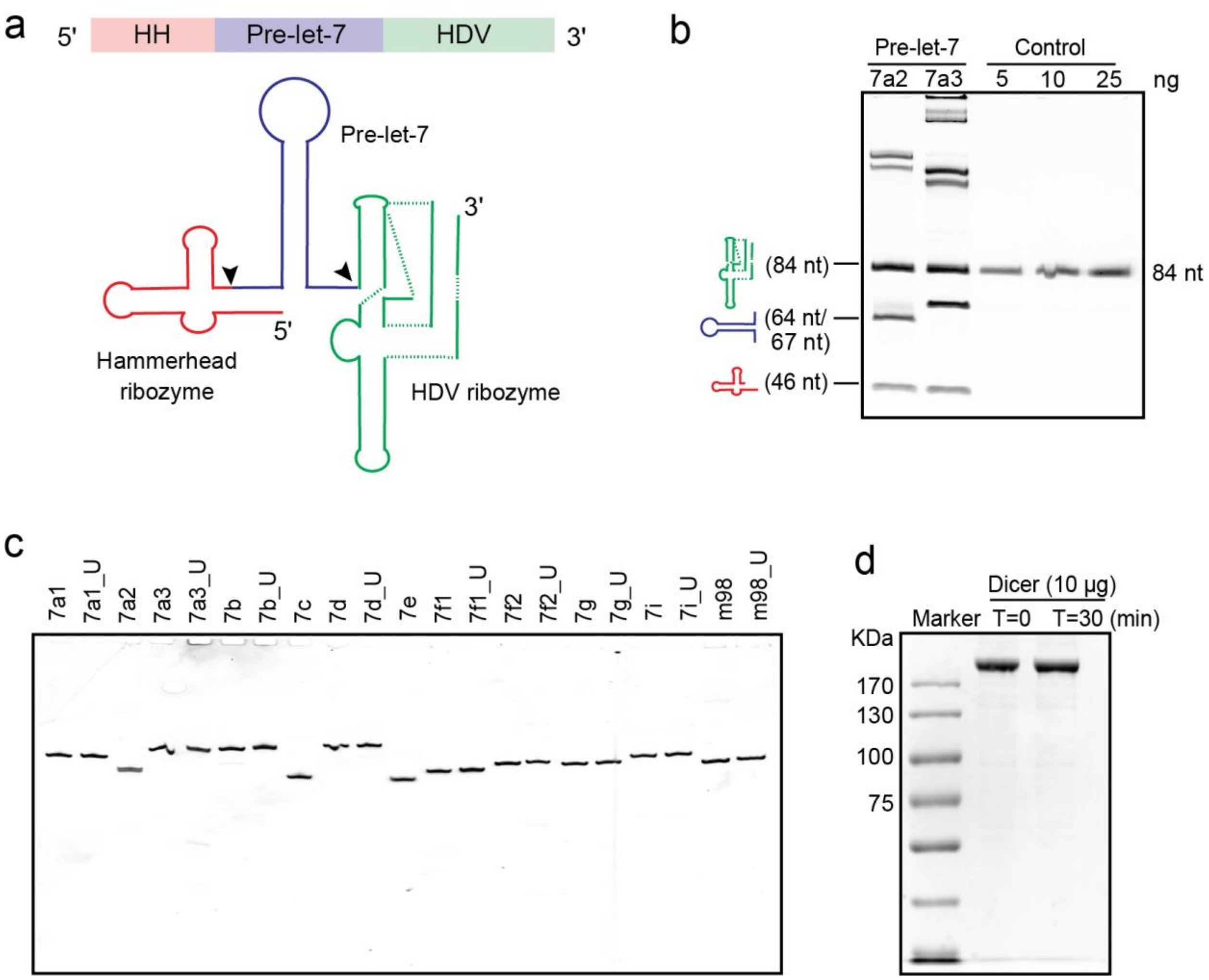

Fig. S3 Quality assurance of purified RNA substrates and enzyme for this study. **a** Schematic representation of a typical T7 RNA transcript containing the pre-let-7 substrate flanked by the hammerhead (HH) and Hepatitis delta virus (HDV) ribozymes. The HH ribozyme is engineered for each pre-let-7 miRNA and added to the 5' end, whereas the HDV ribozyme is attached to the 3' end [3]. **b** Self-cleavage of the T7 RNA transcript into smaller fragments during *in vitro* transcription. The smaller RNA fragments (pre-let-7 RNA, HH ribozyme, and HDV ribozyme) are separated by electrophoresis using a 10% denaturing polyacrylamide gel, and the pre-let-7 RNA is purified, as described in the Material and Methods section. The 84-nt RNA control on the gel is a purified version of the HDV ribozyme. **c** Purified pre-let-7 substrates with homogenous 5' and 3'-ends. **d** SDS-PAGE characterization (7.5% gel) of purified Dicer before and after incubation at 37°C.

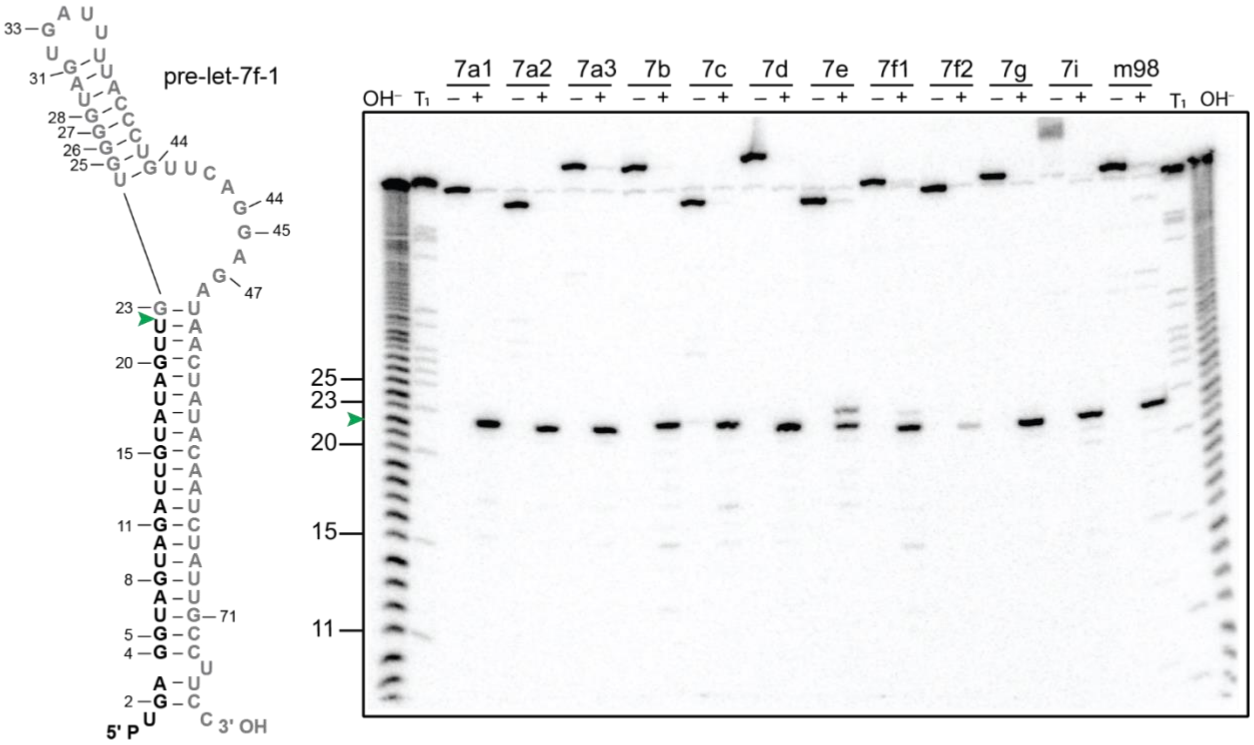

Fig. S4 Characterization of miRNA-5p products for Dicer cleavage of pre-let-7 substrates. Cleavage assays for 12 pre-let-7 RNAs were performed with (+) and without Dicer (-) under single-turnover conditions with 0.1 nM substrate and 5 nM Dicer for 15 min at 37 ºC. The cleavage reactions along with the T_1 and OH^- ladders generated from pre-let-7f-1 were analyzed by denaturing PAGE (right panel). The green arrowhead points to the standard Dicer cleavage site (22-nt). To help identify RNase T1 cleavage products on the denaturing gel**,** the primary and secondary structures of pre-let-7f-1 are shown (left panel) with the G residues numbered according to their position in the sequence.

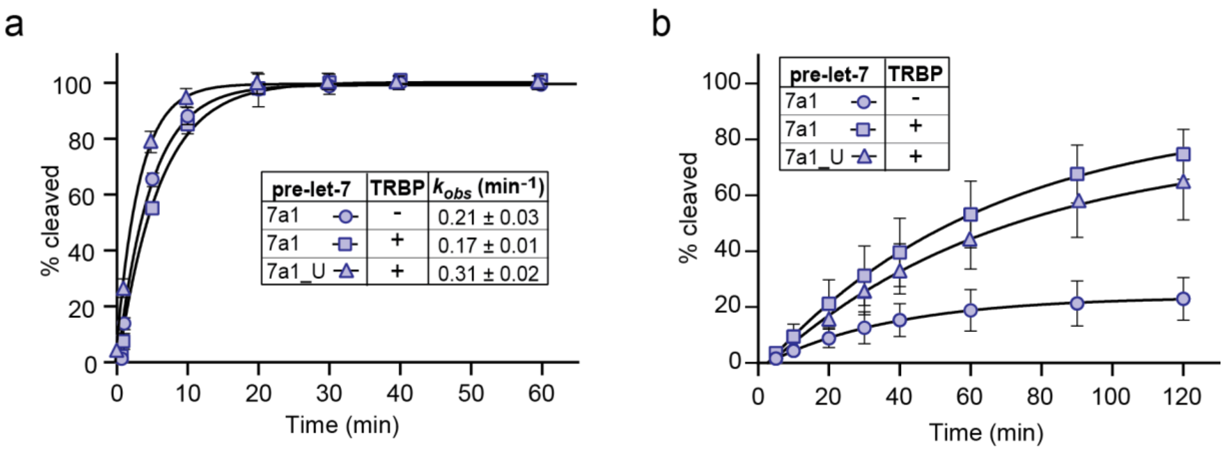

Fig. S5 Effect of TRBP on pre-let-7 cleavage by Dicer. **a** Cleavage assays for pre-let-7a-1 and pre-let-7a-1_U were performed under single turnover conditions (0.1 nM substrate and 5 nM Dicer) in the absence and/or presence of TRBP (5 nM), and the k_{obs} values were derived with standard deviation (shown by the error bars) from the exponential fits of the pseudo-first order cleavage data from at least three independent experiments. **b** Cleavage assays for pre-let-7a-1 and pre-let-7a-1_U under multiple-turnover conditions (25 nM substrate and 0.5 nM Dicer) in the absence and/or presence of TRBP (10 nM). The standard deviations (shown by the error bars) were obtained from at least three independent experiments. In (**a**) and (**b**), the following symbols are used for cleavage under different conditions: circles for pre-let-7a-1 without TRBP; squares for pre-let-7a-1 with TRBP; and triangles for pre-let-7a-1_U with TRBP.

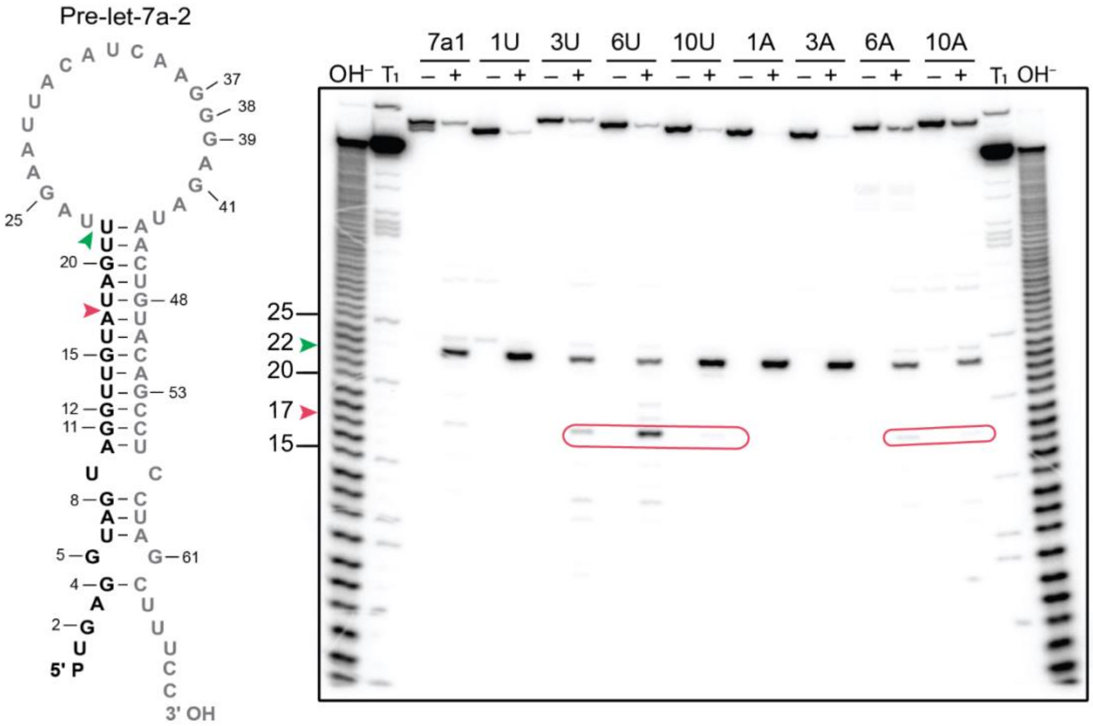

Fig. S6 Characterization of miRNA-5p products for Dicer cleavage of uridylated and adenylated pre-let-7a-1 substrates**.** Cleavage assays for pre-let-7a-1, uridylated pre-let-7a-1, and adenylated pre-let-7a-1 were performed with (+) and without Dicer (-) under single-turnover conditions (0.1 nM substrate and 5 nM Dicer) for 15 min at 37ºC. The cleavage reactions along with the T_1 and OH^- ladders generated from pre-let-7a-2 were analyzed by denaturing PAGE (right panel). The green arrowhead points to the standard Dicer cleavage site (22-nt) and the red arrow indicates the alternative Dicer cleavage site (17 nt). To help identify RNase T1 cleavage products on the denaturing gel**,** the primary and secondary structures of pre-let-7a-2 are shown (left panel) with the G residues numbered according to their position in the sequence.

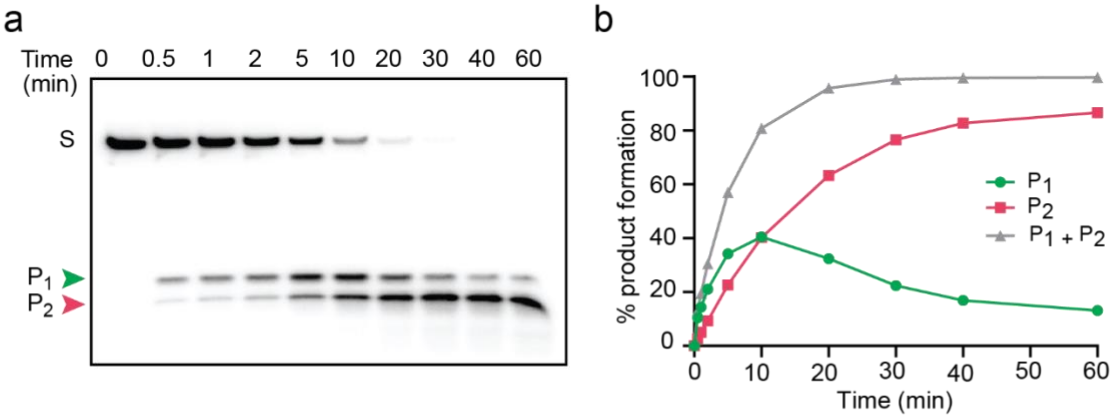

Fig. S7 Time course for Dicer cleavage of pre-let-7a-1_6U under single-turnover conditions. **a** The cleavage assay for pre-let-7a-1_6U was performed under single-turnover conditions using 0.1 nM RNA and 5 nM Dicer. The aliquots were analyzed on 15% denaturing PAGE. The two products, P_1 and P_2, are indicated with green and red arrowheads, respectively. **b** The percentage of product formation at several time-points were determined for both the P_1 (green) and P_2 (red) products individually and for the sum of products (P1 + P2: in grey). The exponential fit (not shown) of the single-turnover kinetic data for substrate cleavage (P_1 + P_2) yields the values of k_{obs} (0.17 min^{-1}) and maximum percentage of cleavage (96.2 %) with an R^2 value of 0.998.

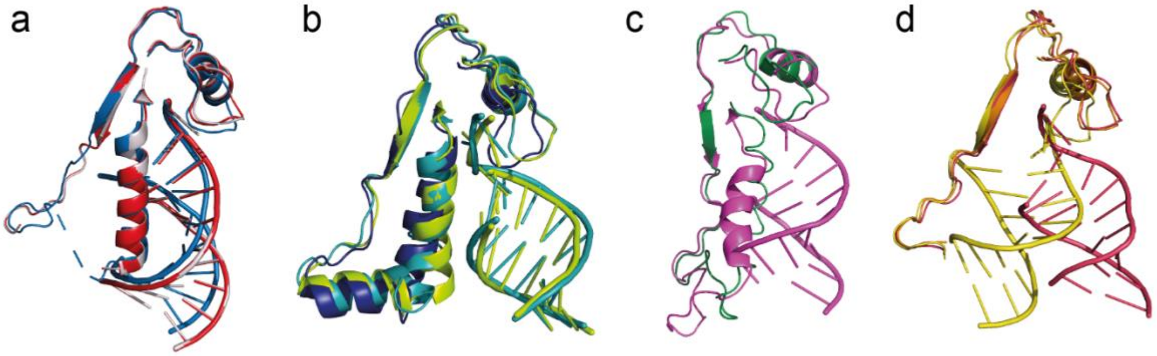

Fig. S8 Plasticity of the PAZ domain towards different substrates. **a** Alignment of crystal structures of the platform-PAZ-connector (PPC) domain of human Dicer in complex with different siRNAs. PDB entries 4NGB (PPC with 12-mer siRNA having UU-3' ends, red), 4NGC (PPC with 12-mer siRNA having UA-3' ends, silver) and 4NGF (PPC with 17-mer siRNA, blue) are shown [4]. **b** Alignment of the PPC domain from the cryo-EM structures of human Dicer free and in complex with a pre-let-7a1 variant in a pre-dicing state. PDB entries 5ZAK (dark blue, Dicer only), 5ZAL (cyan, Dicer-RNA complex with a full RNA stem) 5ZAM (green-yellow, Dicer-RNA complex with a partial RNA stem) are shown [5]. **c** Alignment of the PPC domain from the cryo-EM structures of human Dicer free and in complex with pre-let-7a1^{GYM}. PDB entries 7XW3 (green, Dicer only) and 7XW2 (pink, Dicer-RNA complex in a dicing state) are shown [6]. **d** Alignment of PPC domain from the cryo-EM structures of mouse Dicer in complex with pre-miR-15a. PDB entries 7YZ4 (orange, Dicer only), 7YYM (raspberry, pre-dicing state), and 7YYN (yellow, Dicing state) are shown [7]. The platform-PAZ-connector (PPC) were aligned for all structures and the regions of interaction between the PAZ and RNA substrates are shown.

References

1 Heo I, Ha M, Lim J, Yoon MJ, Park JE, Kwon SC, Chang H & Kim VN (2012) Mono-uridylation of pre-microRNA as a key step in the biogenesis of group II let-7 microRNAs. Cell 151:521-532 https://doi.org/10.1016/j.cell.2012.09.022

2 Zuker M (2003) Mfold web server for nucleic acid folding and hybridization prediction. Nucleic Acids Res 31:3406-3415 https://doi.org/10.1093/nar/gkg595

3 Avis JM, Conn GL & Walker SC (2012) Cis-acting ribozymes for the production of RNA in vitro transcripts with defined 5' and 3' ends. Methods Mol Biol 941:83-98 https://doi.org/10.1007/978-1-62703-113-4_7

4 Tian Y, Simanshu DK, Ma JB, Park JE, Heo I, Kim VN & Patel DJ (2014) A phosphate-binding pocket within the platform-PAZ-connector helix cassette of human Dicer. Mol Cell 53:606-616 https://doi.org/10.1016/j.molcel.2014.01.003

5 Liu Z, Wang J, Cheng H, Ke X, Sun L, Zhang QC & Wang H-W (2018) Cryo-EM structure of human Dicer and its complexes with a pre-miRNA substrate. Cell 173:1191-1203.e1112 https://doi.org/https://doi.org/10.1016/j.cell.2018.03.080

6 Lee Y-Y, Lee H, Kim H, Kim VN & Roh S-H (2023) Structure of the human DICER–pre-miRNA complex in a dicing state. Nature https://doi.org/10.1038/s41586-023-05723-3

7 Zapletal D, Taborska E, Pasulka J, Malik R, Kubicek K, Zanova M, Much C, Sebesta M, Buccheri V, Horvat F, Jenickova I, Prochazkova M, Prochazka J, Pinkas M, Novacek J, Joseph DF, Sedlacek R, Bernecky C, O'Carroll D, Stefl R & Svoboda P (2022) Structural and functional basis of mammalian microRNA biogenesis by Dicer. Mol Cell 82:4064-4079.e4013 https://doi.org/10.1016/j.molcel.2022.10.010
